# Transcriptome profiling of granulosa cells of bovine ovarian follicles during growth from small to large antral sizes

**DOI:** 10.1186/1471-2164-15-24

**Published:** 2014-01-14

**Authors:** Nicholas Hatzirodos, Helen F Irving-Rodgers, Katja Hummitzsch, Margaret L Harland, Stephanie E Morris, Raymond J Rodgers

**Affiliations:** 1 Research Centre for Reproductive Health, Discipline of Obstetrics and Gynaecology, School of Paediatrics and Reproductive Health, Robinson Institute, University of Adelaide, Adelaide, SA 5005, Australia; 2 Current Address: School of Medical Science, Griffith University, Gold Coast, Queensland, Australia

**Keywords:** Ovary, Microarray analysis, Bovine, Granulosa cells, Follicles

## Abstract

**Background:**

At later stages of folliculogenesis, the mammalian ovarian follicle contains layers of epithelial granulosa cells surrounding an antral cavity. During follicle development granulosa cells replicate, secrete hormones and support the growth of the oocyte. In cattle, the follicle needs to grow > 10 mm in diameter to allow an oocyte to ovulate, following which the granulosa cells cease dividing and differentiate into the specialised cells of the corpus luteum. To better understand the molecular basis of follicular growth and granulosa cell maturation, we undertook transcriptome profiling of granulosa cells from small (< 5 mm; n = 10) and large (> 10 mm, n = 4) healthy bovine follicles using Affymetrix microarrays (24,128 probe sets).

**Results:**

Principal component analysis for the first two components and hierarchical clustering showed clustering into two groups, small and large, with the former being more heterogeneous. Size-frequency distributions of the coefficient of variation of the signal intensities of each probe set also revealed that small follicles were more heterogeneous than the large. IPA and GO enrichment analyses revealed that processes of axonal guidance, immune signalling and cell rearrangement were most affected in large follicles. The most important networks were associated with: (A) Notch, *SLIT/ROBO* and *PI3K* signalling, and (B) *ITGB5* and extracellular matrix signalling through extracellular signal related kinases (ERKs). Upstream regulator genes which were predicted to be active in large follicles included *STAT* and *XBP1.* By comparison, developmental processes such as those stimulated by *KIT*, *IHH* and *MEST* were most active in small follicles. *MGEA5* was identified as an upstream regulator in small follicles. It encodes an enzyme that modifies the activity of many target proteins, including those involved in energy sensing, by removal of N-acetylglucosamine from serine and threonine residues.

**Conclusions:**

Our data suggest that as follicles enlarge more genes and/or pathways are activated than are inactivated, and gene expression becomes more uniform. These findings could be interpreted that either the cells in large follicles are more uniform in their gene expression, or that follicles are more uniform or a combination of both and that additional factors, such as LH, are additionally controlling the granulosa cells.

## Background

An ovarian primordial follicle is composed of an inactive oocyte surrounded by granulosa cells all enclosed by a basal lamina. Once activated the follicle grows by enlargement of the oocyte and replication of the granulosa cells from about 24 cells to 50 million cells in the cow [1]. During growth, a fluid-filled antrum or cavity also develops in the middle of the follicle [2] and bovine follicles need to enlarge to over 10 mm in diameter, principally by antrum expansion, to be capable of ovulation. Ovulation occurs only once per oestrous cycle. However, instead of one primordial follicle growing to the necessary size and then ovulating, many follicles commence growth during the course of the cycle. The vast majority of these growing follicles become atretic leaving in cows only one, or occasionally two, follicles to ovulate. The process of follicle growth during a cycle is not random either, since two or three groups or waves of follicles emerge from a pool of follicles of approximately 5 mm in diameter during each oestrous cycle [3,4]. During these maturational waves, follicles continue to enlarge over several days until one follicle that is growing faster and is hence larger than the others gains dominance [5,6]. Thus a deviation in the size of follicles occurs when they are around 7–8 mm in diameter [7]. As the larger dominant follicle continues to expand further, the smaller follicles in the wave undergo atresia. If the wave is at the end of a cycle the dominant follicle ovulates and a new cycle is initiated. In earlier waves the dominant follicle also eventually undergoes atresia and another wave then ensues.

During growth of the follicle, the granulosa cells undergo a number of maturational changes. Early in follicle development they secrete the hormone inhibin and later at the pre-ovulatory sizes, oestradiol. The cells also express follicle-stimulating hormone receptors soon after follicle activation and then during the course of dominance they additionally express luteinising hormone receptors (LHCGR). The process of dominance is not well understood largely because it is not possible to trace the cellular changes that occur within a follicle in real time in order to relate the events preceding development to future outcomes, such as predicting whether an individual follicle will become dominant or subordinate. Another recent approach compared identical-sized follicles before deviation into dominant and subordinate follicles and analysed gene expression [8]. In that study a firm hypothesis was investigated and it was found that follicles with the highest level of *CYP11A1*, encoding the rate limiting enzyme for progesterone synthesis, also had the highest level of *CYP19A1*, encoding the rate-limiting enzyme for oestradiol synthesis [8]. It also had the highest expression level of three genes (*LAMB2*, *COL4A1*, *HSPG2*), encoding components of an unusual basal lamina matrix, focimatrix (abbreviated from focal intra-epithelial matrix) [8]. It was concluded that since these five genes continue to be further up regulated in dominant and preovulatory-size follicles and because the expression levels of these genes were correlated with each other, that focimatrix production and *CYP11A1* expression might be important in a follicle gaining dominance [8].

Focimatrix develops as aggregates of basal lamina material deposited between the granulosa cells and contains the α1 and α2 chains of collagen type IV, laminin α1, β2 and γ1 chains, nidogen-1 and −2, perlecan, collagen type XVIII and usherin, but not versican [9]. These components are similar to those found in the follicular basal lamina at the stage of follicular development when focimatrix is first observed [10,11]. Focimatrix initially appears in bovine follicles greater than 5 mm in diameter, and the amount of focimatrix increases with increasing follicular size [9]. This first appearance of focimatrix occurs as follicles emerge in a growth wave, and prior to emergence of the dominant follicle.

The aim of this study, therefore, was to identify the important processes occurring at the key stages of antral follicle development at the time 1) prior to follicles entering a wave and 2) prior to ovulation, by gene expression array profiling. In order to gain a greater knowledge of the mechanisms responsible for granulosa cell maturation and selection of dominant follicles there have been several transcriptome analyses of bovine granulosa cells [12-17]. Evans and colleagues [12] examined dominant and subordinate follicles (some of which were atretic) by two-color hybridisation on a self -generated array containing approximately 1,300 putative genes. Serial Analysis of Gene Expression (SAGE) tags were examined in follicles of a larger size (8 mm) around the time of deviation for selection of the dominant follicle [13]. Skinner et al. [14] isolated healthy antral follicles at three different sizes, and used pooled follicle RNA to hybridise to individual arrays. Liu et al. [15] was also interested in selection of the dominant follicle using a two color array, but did not separate the granulosa and thecal compartments for analysis. Subordinate, dominant and preovulatory follicles have also been examined by RNA-seq and the effects of lactation examined on gene expression pathways [16]. More recently, Christenson et al. [17] also used microarray analysis to investigate gene expression in bovine antral follicles before and after the LH surge. Only in one of these studies were comparisons made between small follicles, less than 5 mm in diameter, and larger follicles, but the analysis may have been compromised by a lack of statistical power (n = 2/ group). Smaller follicles represent those before focimatrix is expressed and before follicles have entered a wave. Hence we chose to compare these smaller follicles with larger preovulatory-size follicles; all of which were validated as healthy. Additionally we ensured that the isolated granulosa cells were devoid of any potentially contaminating theca cells.

## Results and discussion

### Selection of follicles for analyses

**Figure 1:**
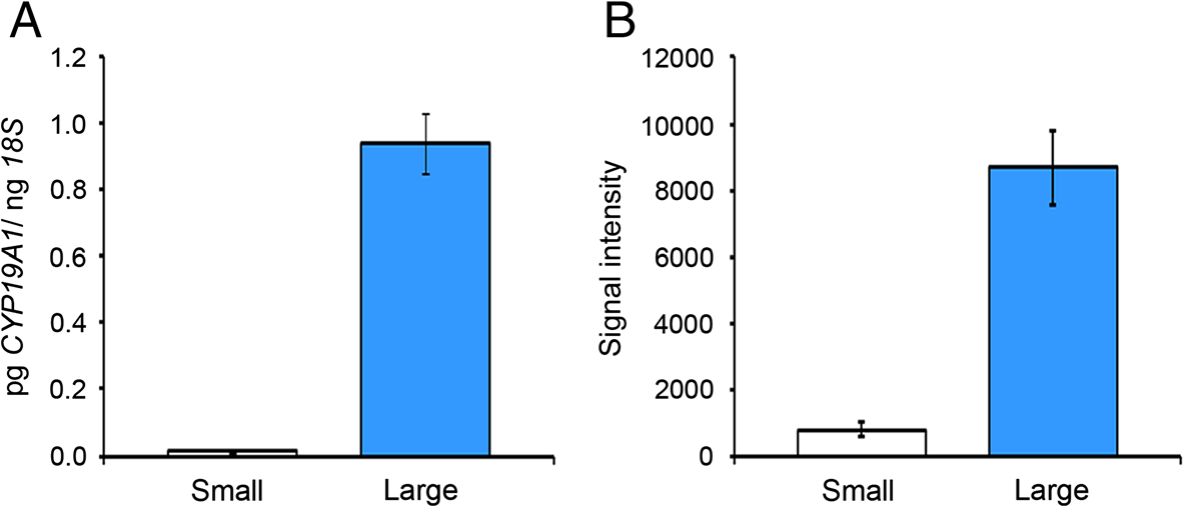
**Quantitative RT-PCR analysis of CYP19 expression (A) compared with gene induction profiles observed by microarray analysis (B).** Mean ± SEM expression of *CYP19* was determined in granulosa cells derived from the 10 small (clear columns) and 4 large follicles (blue columns) used for the microarray analysis. Microarray signal data were non-log transformed and divided by 10^4^.

To ensure accurate comparisons were made between granulosa cells from small (3.2 ± SEM 0.2 mm in diameter; n = 10) versus large (15.3 ± 0.6 mm; n = 4) follicles, only antral follicles of healthy morphology [18,19] were selected for this study. Confirmation of health stage was also performed on large follicles showing *CYP19A1* expression assessed by qRT-PCR similar to that observed in healthy large follicles using microarray analysis (Figure 1) [20]. To ensure that the isolated granulosa cells were not contaminated with any thecal cells the level of *CYP17A1* was measured. *CYP17A1* is expressed exclusively in thecal cells [21]. No follicles with more than 1% level of expression of *CYP17A1* found in thecal samples were included in the analysis. Since there were some low yields of RNA, three of the samples of small follicles were pools of two follicles, each from the same animal.

### Validation of microarray data

To confirm changes in the expression of genes identified by microarray analysis, quantitative RT-PCR analyses of *CYP19A1* were performed on the same samples. Similar to that observed in microarray analysis, the expression of *CYP19A1* was significantly increased in granulosa cells isolated from large follicles compared to granulosa cells isolated from small follicles (Figure 1). The microarray analyses also identified genes well known to be up regulated across the sizes of follicles examined. Some examples of these include hormone-related genes *CYP11A1* (increased 5.8 fold), *HSD3B1* (6.0 fold), *LHCGR* (8.8 fold) and *INHBA* (3.8 fold) and focimatrix genes *COL4A1* (6.8 fold) and *LAMA1* (5.8 fold) as shown previously [20,22] (Table 1).

**Table 1:** **Genes which are up regulated in large follicles with respect to small follicles**†

**Gene symbol**	**Fold change**	**Gene symbol**	**Fold change**	**Gene symbol**	**Fold change**
**Cell cycle**					
*RASA2*	5.5	*TACC1*	3.5	*RAD50*	3.1
*TBCEL*	4.8	*TOPORS*	3.3	*HELZ*	3.1
*CDK13*	4.3	*TRIB2*	3.2		
*TPR*	4.3	*ANAPC5*	3.1		
**Cell morphology**					
*LIMA1*	8.2	*FERMT2*	4.2	*ARPC1B*	3.3
*SLMAP*	5.1	*TTLL3*	3.9		
*MYO1B*	5.1	*ABLIM1*	3.7		
**Cytokines, hormones and receptors**					
*IFI30*	39.0	*F2RL1*	5.3	*THBS3*	3.5
*PTHLH*	12.8	*DKK3*	5.3	*IGFBP6*	3.5
*F2R*	8.9	*NPR3*	5.2	*BMPR2*	3.3
*LHCGR*	8.8	*LPHN2*	4.7	*BAI2*	3.3
*IGFBP4*	7.3	*PGR*	4.7	*GPR88*	3.2
*F3*	7.2	*TNFAIP8L3*	4.7	*SCG2*	3.2
*OPTN*	6.5	*GABARAPL1*	4.6	*OSBPL8*	3.1
*IL4R*	6.2	*STRA6*	4.2	*TM2D1*	3.1
*IL6R*	5.7	*RYK*	4.2	*BMPR1A*	3.1
*GRK5*	5.6	*INHBA*	3.8	*NRP1*	3.0
*NR5A2*	5.5	*LGALS3BP*	3.5	*GPR173*	3.0
*PTGFR*	5.5				
**Directional cell growth**					
*LRP8*	53.6	*SLITRK2*	5.1	*SEMA6A*	3.3
*EFNA5*	8.6	*PLXNC1*	4.8	*NINJ1*	3.2
*MPP5*	7.1	*PLXNB2*	4.7	*ROBO1*	3.1
*ROBO2*	6.7	*EPHB6	3.8	*ROCK1*	3.1
*SEMA6D*	6.2	*COLEC11*	3.5		
**Extracellular matrix and synthesis**					
*TNFAIP6*	279.6	*COL16A1*	6.0	*VCAN*	5.0
*SPOCK2*	22.0	*LAMA1*	5.8	*LEPREL1*	4.9
*COL4A1*	6.8	*PCOLCE*	5.7	*SDC2*	3.8
**Intercellular and cell to matrix adhesion**					
*ARHGAP18*	20.5	*CDH11*	4.6	*TSPAN9*	3.4
*ITGB5*	11.4	*BST2	4.4	*PARD3B*	3.2
*VCAM1*	7.3	*TNS3*	3.6	*TSPAN2*	3.0
*CSPG4*	5.5	*CCM2*	3.6	*UBQLN1*	3.0
*ARHGAP17*	4.8				
**Ion transport**					
*TMEM20	4.3				
**Protein trafficking**					
*HSP90AA1*	7.0	*SH3GL2*	4.1	*RAB7A*	3.3
*TMEM27*	5.1	*SCYL2*	4.0	*CLGN*	3.2
*GOLGA4*	4.6	*MLEC*	3.8	*CLTC*	3.2
*TMEM47*	4.5	*PLEKHG1*	3.6	*FKBP9*	3.1
*PLEKHA2*	4.2	*PLEKHB2*	3.5	*CANX*	3.1
*PLEKHH3*	4.1	*GDI1*	3.4		
**Proteolysis or inhibition**					
*PRSS23*	48.5	*ADAMTS4*	4.7	*FBXL20*	3.9
*PLAT*	17.5	*HM13*	4.5	*ADAM10*	3.7
*ACE2*	12.9	*RNF128*	4.5	*MYCBP2*	3.4
*CPD*	11.8	*CTSB*	4.4	*HERPUD1*	3.3
*SERPINA5*	11.2	*SPG7*	4.2	*RNF20*	3.3
*ECE1*	9.5	*DERL1*	4.1	*UBR3*	3.2
*ADAM9*	8.4	*USP4*	4.0	*MARCH5*	3.2
*TIMP2*	7.9	*UBR1*	4.0	*FAF2*	3.2
*ADAM12*	6.6	*MARCH6*	3.9	*TTC3*	3.1
*USP7*	6.4				
**RNA processing**					
*NOL3*	6.0	*PBRM1*	4.3	*LUC7L3*	3.8
*CPEB4*	5.1	*PRPF38B*	4.2	*PNISR*	3.7
*UTP6*	4.8	*RBM5*	4.0	*SYNCRIP*	3.6
*CSDE1*	4.7	*TIA1*	3.9	*RNASEK*	3.6
*CHD1*	4.7	*CTR9*	3.9	*DDX46*	3.4
*SF3B1*	4.6	*HNRPLL*	3.9	*DICER1*	3.1
*RBM25*	4.4				
**Transcription regulation**					
*TOX*	11.4	*ZNF317*	3.8	*BRWD1*	3.3
*NMI*	8.4	*HUWE1*	3.8	*NOSTRIN*	3.2
*NOTCH1*	8.4	*TRIM25*	3.7	*JARID2*	3.2
*TOB1*	6.3	*CREB3L2*	3.7	*ZNF462*	3.1
*ZNF292*	6.0	*ADNP*	3.7	*ANKRD10*	3.1
*MTPN*	5.7	*ZNF609*	3.6	*CITED2*	3.1
*AFF1*	5.5	*KLF6*	3.5	*MED24*	3.1
*FOXP2*	5.3	*MLL3	3.5	*GPS2*	3.1
*ID2*	4.5	*NCOR1*	3.5	*HMGXB3*	3.1
*ID3*	4.3	*RBFOX2*	3.4	*TFCP2*	3.1
*SON	4.2	*ZNF24*	3.3	*CITED1*	3.1
*WHSC1L1*	3.9				
**Translation regulation**					
*EIF4G3*	7.4	*BZW2*	3.6	*EIF2C2*	3.3
*EIF4EBP1*	7.3	*EIF2C3*	3.4		
*BZW1*	4.2	*IREB2*	3.4		
**Transport**					
*SLC5A11*	15.6	*MAL2*	3.8	*SEC63*	3.4
*APOA2*	12.5	*CLIC4*	3.7	*STAR*	3.4
*SLC39A8*	9.1	*CPNE8*	3.7	*TFR2*	3.3
*AP2B1*	6.8	*ATP13A3*	3.6	*COPZ1*	3.3
*SLC25A28*	5.8	*SLC25A12*	3.6	*MICU1*	3.2
*SLC27A3*	5.7	*SLC26A11*	3.6	*NUP85*	3.2
*TNPO1*	5.5	*AP3S2*	3.6	*CYCS*	3.2
*SLC40A1*	5.3	*TM9SF4*	3.5	*APOA1*	3.2
*ABCB1*	5.1	*AP1S2*	3.5	*CLTC*	3.2
*ATP6V1A*	4.3	*ACBD5*	3.5	*STBD1*	3.1
*RRBP1*	4.2				
**Other enzymes**					
*ME3*	26.7	*PTPN13*	4.2	*GPX3*	3.2
*CYBB*	18.1	*SCD5*	4.2	*PDP1*	3.2
*CYP19A1*	14.2	*FDFT1*	4.1	*LARGE*	3.2
*NT5E*	12.8	*UGCG*	4.0	*MLL5*	3.2
*RGN*	9.8	*DPYSL2*	3.8	*EDEM2*	3.1
*PYGL*	8.9	*PLD1*	3.7	*NCEH1*	3.1
*B3GALT2*	6.5	*PPAP2B*	3.7	*XRN2*	3.1
*PDSS1*	6.0	*TET2*	3.7	*RDH11*	3.1
*HSD3B2*	6.0	*PFKM*	3.7	*CHST10*	3.1
*PPM1K*	5.9	*SGSH*	3.6	*PTP4A2*	3.1
*CYP11A1*	5.8	*CUL3*	3.5	*PTPN11*	3.1
*PIGS*	5.5	*QSOX1*	3.5	*MAOA*	3.1
*IDH3A*	5.5	*ACSS2*	3.5	*CA8*	3.0
*CMAHP*	4.6	*DPYD*	3.4	*HECTD1*	3.0
*PDPK1*	4.5	*MTR*	3.4	*PPP2R5E*	3.0
*AHCYL2*	4.4	*POR*	3.3	*CUL1*	3.0
**Other signalling**					
*BEX2*	9.0	*ARHGEF3*	4.8	*PRKAG2	3.4
*PIK3R1*	9.0	*AMIGO2*	4.7	*SNTB2*	3.4
*DTNA*	8.5	*MAP2K4*	4.4	*GLG1*	3.3
*DACT1*	8.4	*NDRG3*	4.4	*GNA11*	3.3
*ARFGAP3*	7.6	*APC*	4.0	*IER3*	3.3
*GADD45B*	6.8	*PDCD4*	4.0	*NISCH*	3.3
*NDRG4*	6.7	*BMP2K*	3.8	*INSIG2*	3.2
*BCAS3*	6.0	*Ifi27l1*	3.7	*STIM1*	3.2
*MIA3*	5.4	*ERRFI1*	3.6	*BCL2L2*	3.2
*MS4A8B*	5.3	*ARHGEF6*	3.5	*PIP4K2A*	3.1
*SAFB2*	4.9	*FICD*	3.4	*KIRREL*	3.0
*MAPK6*	4.8				
**Other**					
*FAM114A1*	18.8	*TMEM176A*	4.1	*ANKRD12*	3.4
*DDX26B*	9.7	*WDFY4*	3.9	*WDFY2	3.4
*LHFPL2*	6.8	*PSMD4*	3.8	*CHCHD10*	3.3
*RNF213*	5.9	*RHBDD2*	3.8	*MRAP*	3.3
*OBSL1*	5.8	*ZNF317	3.8	*TMEM50B*	3.2
*USHBP1*	5.2	*TMEM176B*	3.7	*BRWD3*	3.2
*RSRC2*	5.1	*PSAP*	3.7	*OXR1	3.2
*KLHL28*	5.0	*LRIG3*	3.7	*HIATL1*	3.1
*LINGO2*	5.0	*AKAP8L*	3.6	*MGARP*	3.1
*PDLIM4*	5.0	*FAM126B*	3.6	*PHF3*	3.1
*ODF2L*	4.4	*RCN3*	3.6	*MPV17L2*	3.1
*BTBD7*	4.3	*LRRC2*	3.6	*TBC1D5*	3.0
*RNF144B*	4.3	*SUSD4*	3.5	*Gm16462/*	3.0
*R3HCC1*	4.2	*UHRF1BP1L*	3.5	*Gm8787*	
*TXNIP*	4.1	*YPEL5*	3.5	*VWA1*	3.0
*RCAN3*	4.1	*FAM174B*	3.4	*ZBTB33*	3.0

^†^(≥3 fold, *P* < 0.05) and categorised by function. Genes are listed in descending order of fold change within each category. Significance was determined by Benjamini-Hochberg post-hoc test for multiple corrections following one way ANOVA.

*indicates genes determined from the Partek analysis based on the Affymetrix annotations which were not assigned identities by IPA.

### Statistical analyses of gene expression

**Figure 2:**
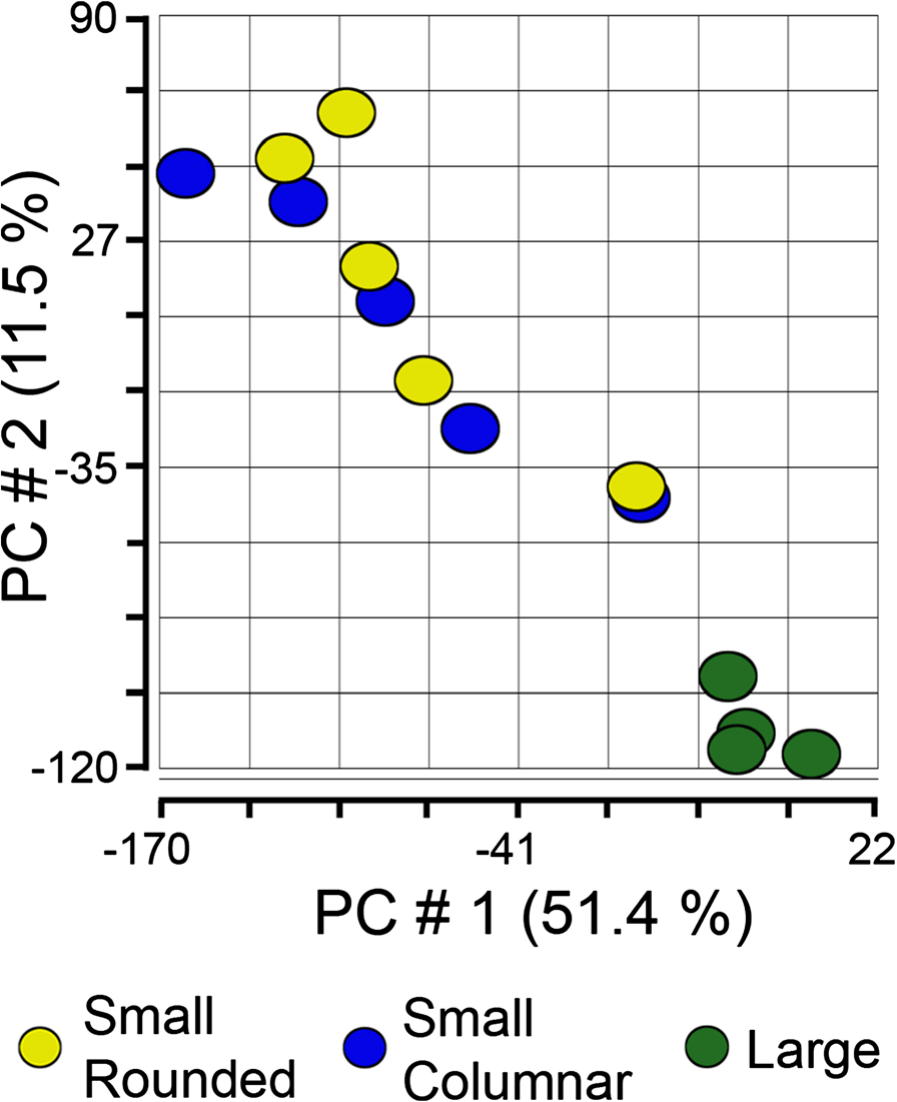
**Unsupervised principal component analysis (PCA) of arrays for small (n = 5 rounded phenotypes in yellow and n = 5 columnar phenotypes in blue) and large (n = 4, in green) follicles in Partek.** The graph is a scatter plot of the values for the first (X) and second (Y) principal components based on the correlation matrix of the total normalised array intensity data.

Granulosa from small healthy follicles were from one of two groups having either columnar (n = 5) or rounded (n = 5) shaped basally-situated granulosa cells as described in the Methods. Principal component analysis (PCA) for the first three components (Figure 2) and hierarchical clustering (Additional file 1: Figure S1) for the total number of probe sets (n = 24,182) of all arrays in this study was conducted. Neither of these unsupervised analytical methods separated the small healthy follicle arrays into the rounded and columnar groups, and in fact no genes were shown to be more than 2-fold differentially expressed between the two subgroups with a Benjamini-Hochberg False Discovery Rate (FDR) of *P* < 0.05 by ANOVA. Therefore, the small healthy follicles were treated as a single group for further analyses (n = 10) and compared with the group of large follicles (n = 4). It can be clearly seen that the large follicles clustered comparatively closely together and differed from the small healthy follicles, which appeared to be more variable across the group. This was also reflected in the hierarchical clustering analysis (Additional file 1: Figure S1). Seven hundred and fifty eight probe sets were found to be differentially expressed between small and large follicles, when a *P <* 0.05 and an arbitrary threshold of 3-fold minimum differential expression was applied (Table 2). These consisted of 579 up-regulated and 179 down-regulated probe sets in large with respect to small follicles. The fact that substantially more genes were up regulated than down regulated in large healthy follicles, could indicate that activation rather than a reduction in additional pathways occurs as follicles enlarge.

**Table 2:** Numbers of probe sets 2 fold or more differentially expressed in large healthy follicles with respect to small healthy follicles*

**Fold change**	**Up-regulated**	**Down-regulated**	**Total**
> 2	1666	1048	2714
> 3	579	179	758
> 4	278	67	345

*Significant by FDR with *P* < 0.05, with ANOVA in Partek using the step-up Benjamini-Hochberg FDR method for multiple corrections.

### Variability of gene expression

**Figure 3:**
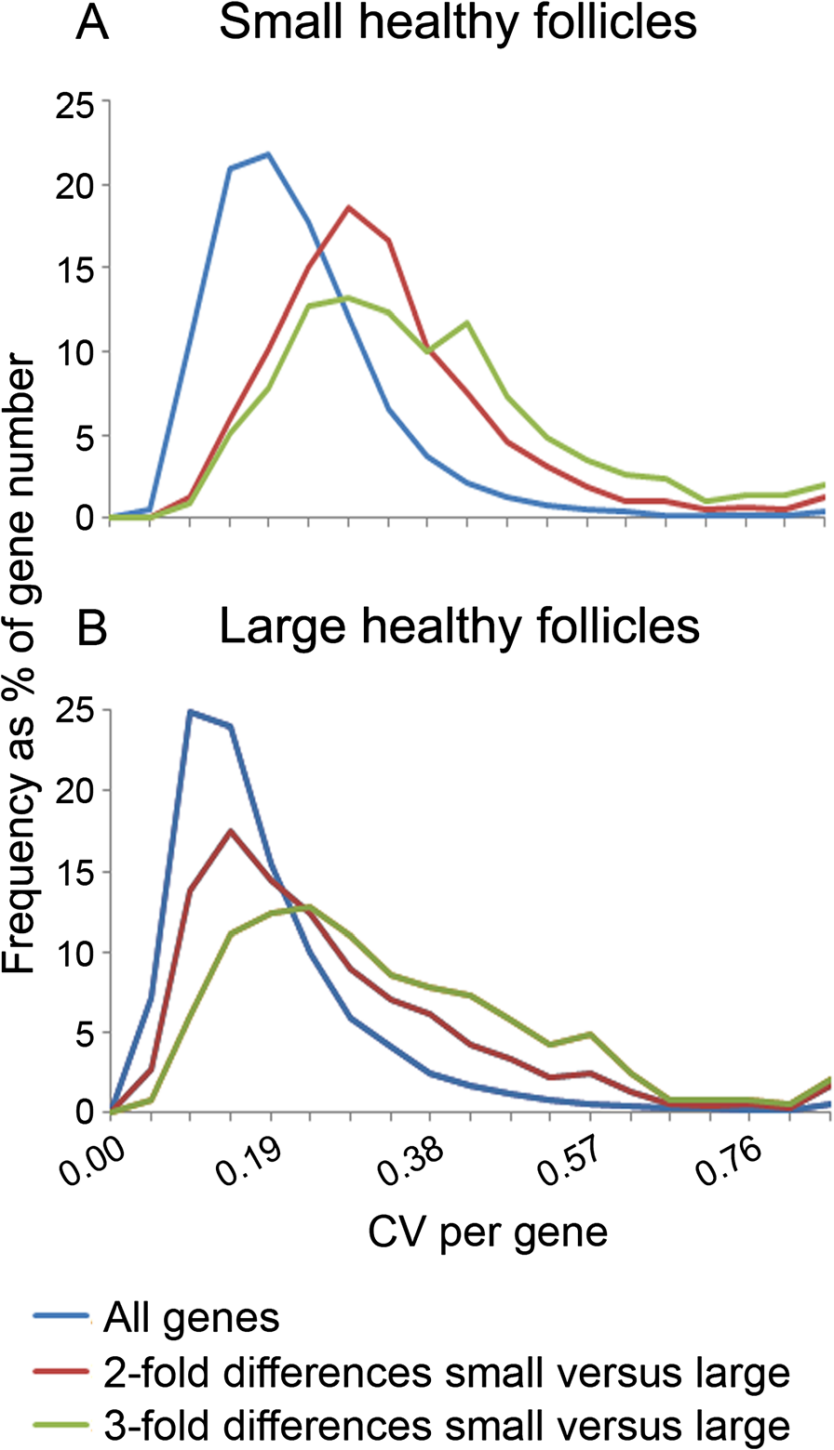
**Plots of coefficients of variation (CV) versus their frequency for granulosa cell cDNA hybridised to Bovine Genome Affymetrix Expression arrays across replicate samples per gene for small (n = 10) in A and large follicles (n = 4) in B.** All genes include all the probe sets present on the array (n = 24,128 ). 2 fold and 3 fold represent all probe sets which were 2-fold (n = 2,780) or 3-fold (n = 760) differentially regulated between small and large follicles in Partek.

The Coefficient of Variation (SD/Mean X 100 = CV) for each gene in small and in large follicles in both the complete probe set and the >2-fold differentially regulated probe set were calculated. The CV-frequency distribution plots are shown in Figure 3. The small follicles (Figure 3A) had more genes that were variably expressed, particularly for the genes whose expression was >2-fold differentially regulated between small and large follicles (Figure 3B). Furthermore, when we repeated the analysis using only the genes whose signal intensities were in the top 50% on the array we still observed a shift to increased variation, thus demonstrating that this property is inherent in the small follicles rather than possibly due to overall lower array intensities (Additional file 2: Figure S2). The higher variability in gene expression in the small follicle granulosa cells indicates that either the cells in small follicles were less uniform in their gene expression, or that small follicles were less uniform or a combination of both. The more variably expressed genes were up regulated during follicle enlargement which indicates that the reduction in variability of gene expression and their up regulation are potentially both important processes in follicle maturation.

### Differentially expressed genes

A list of genes which were differentially regulated between small and large healthy follicles (758 probe sets, 3-fold differentially expressed, *P* < 0.05, gene list and names are shown in Additional file 3: Table S1) was examined in detail. We were able to identify genes which were well characterised during follicle development, and known to be differentially regulated between small and large follicles in our microarray analyses which included *LHCGR*[23], progesterone receptor [24], *INHBA*[25] and the receptor for the lipid mediator prostaglandin F2α (*PTGFR*) [26]. In the list 533 probe sets could be assigned gene identities in Ingenuity Pathway Analysis (IPA) which included those with homology to multiple probe sets, consisting of 446 annotated genes, of these 352 were up regulated in large follicles (Table 1) and 92 were down regulated (Table 3). This list was also uploaded to the Gene Ontology Enrichment Analysis Software Toolkit (GOEAST) program.

**Table 3:** **Genes which are down regulated in large follicles with respect to small follicles**†

**Gene symbol**	**Fold change**	**Gene symbol**	**Fold change**	**Gene symbol**	**Fold change**
**Cell cycle**					
*RPRM*	5.8				
**Cell morphology**					
*JAKMIP1*	20.8	*MYO10*	6.8	*ACTA1*	3.4
*SEPT4*	15.3	*MFAP2*	4.6	*MYO1D*	3.0
**Cytokines, hormones and receptors**					
*KIT*	23.1	*IL33*	3.6	*GPR77*	3.2
*ANGPT2*	11.5	*PTPRN2*	3.6	*ANGPTL2*	3.1
*RYR2*	7.0	*SHISA2*	3.5	*F2RL2*	3.1
*PDGFC*	4.6	*CMTM8*	3.4	*SFRP4*	3.0
*FGFR2*	3.7	*IL20RA*	3.4		
**Directional cell growth**					
*FEZ1*	3.8	*EPHA1*	3.2		
**Extracellular matrix and synthesis**					
*LAMC2*	3.4	*COL1A1*	3.0		
**Intercellular and cell to matrix adhesion**					
*NEDD9*	5.9	*CDH2*	4.0	*NPNT*	3.2
**Protein trafficking**					
*SNX31*	6.0	*ADCK3*	3.4	*MZB1*	3.1
*CLU*	4.9				
**Proteolysis and inhibition**					
*PTI	5.6	*LTF*	4.0	*EPHX1*	3.4
*MMP16*	4.0	*PRSS35*	3.7	*TRIM2*	3.3
**RNA processing**					
*CPEB1*	3.9				
**Transcription regulation**					
*MYC*	16.4	*FOS*	4.0	*EMX2*	3.1
*HOPX*	13.8	*FHL2*	3.3	*MSX1*	3.1
*TGIF1*	6.1	*HES1*	3.2		
**Transport**					
*SVOPL*	8.6	*NUP210*	4.7	*AQP1*	3.2
*NALCN*	6.0	*ABCC8*	4.7	*FXYD6*	3.2
*AP3B2*	5.5	*STARD10*	3.4	*ZP3*	3.2
**Other enzymes**					
*GATM*	11.1	*MAN1A1*	4.2	*PTGS2*	3.5
*CA14*	8.8	*ENPP1*	4.1	*ABAT*	3.5
*PAPSS2*	7.8	*HMOX1*	4.1	*PFKFB3*	3.2
*RASL11B*	6.8	*PHGDH*	3.7	*CYP2C19*	3.1
*GALNT13*	5.3	*RENBP*	3.6	*AKR1C3*	3.1
*AKR1B1*	4.5	*DDO*	3.6	*GYLTL1B*	3.0
**Other signalling**					
MEST	28.7	*GUCA1A*	5.1	*RGS2	3.4
TNFAIP2	17.5	*HLA-A*	4.4	*APBB2*	3.2
IHH	16.6	*CHRDL1	3.7	*BANK1*	3.1
CARTPT	14.0	*SASH1*	3.5	*TCRA	3.0
**Other**					
*LRRC17*	7.1	*TTN*	3.6	*MMD*	3.1
*STAC3*	3.9	*SEL1L3*	3.6	*BTNL1*	3.1
*LRRC1*	3.8	*CCDC3*	3.2	*TMIGD2*	3.0

^†^(≥ 3-fold, *P* < 0.05) and categorised by function. Genes are listed in descending order of fold change in each category. Significance was determined by Benjamini-Hochberg post-hoc test for multiple corrections following one way ANOVA.

*indicates genes determined from the Partek analysis based on the Affymetrix annotations which were not assigned identities by IPA.

### Pathway and network analyses

**Figure 4:**
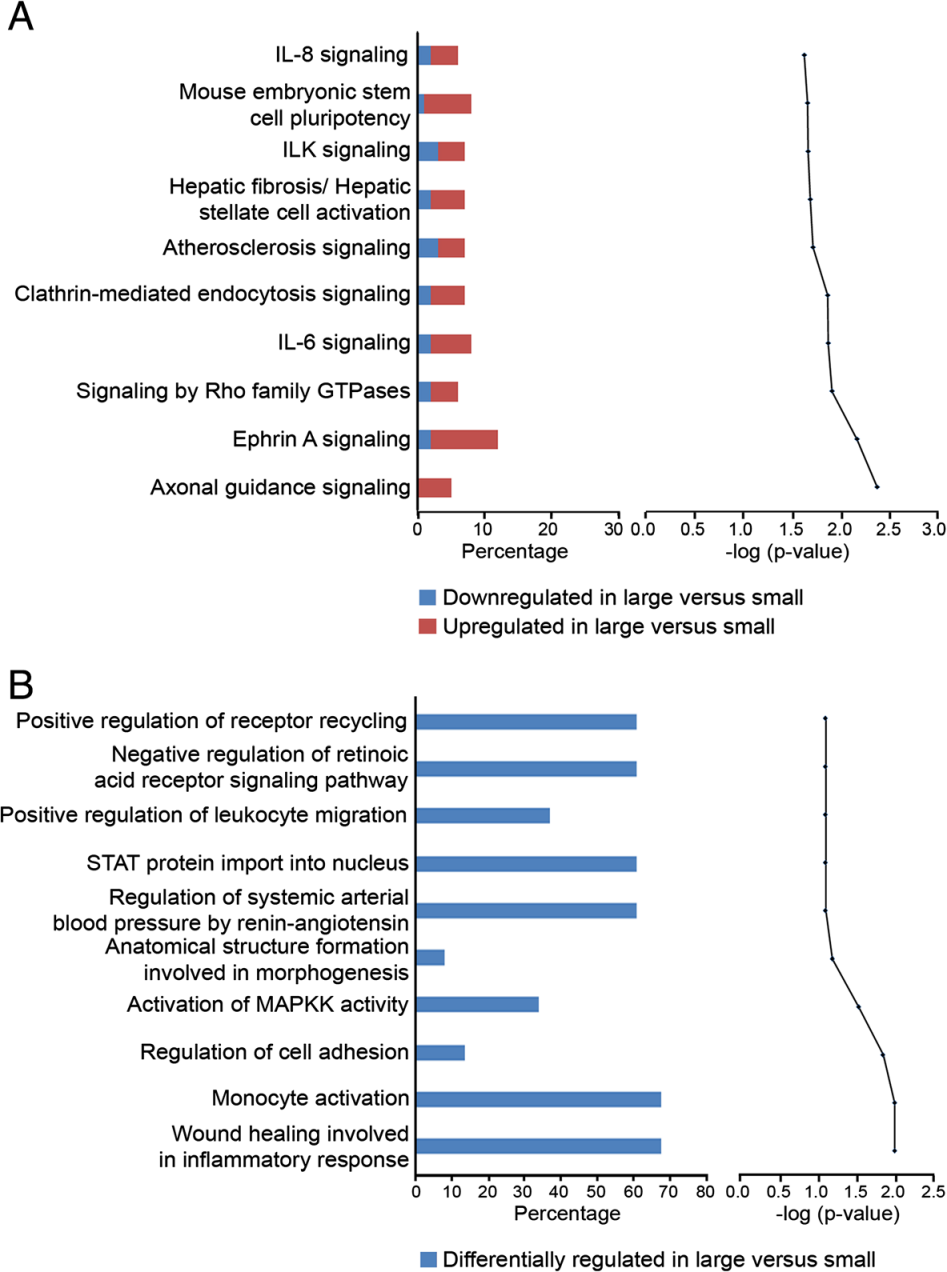
**Top ranked canonical pathways generated in Ingenuity Pathway Analysis (A), and enriched GO terms of interest (B), determined by the GOEAST program, for a set of genes 3-fold differentially regulated with a Benjamini Hochberg False Discovery Rate *P <*** **0.05 between small and large follicles. A** The bar chart on the left represents the percentage of genes from the data set that map to each canonical pathway showing those which are up regulated (in red) and down regulated (in blue) in large with respect to small follicles. The pathways are ranked from lowest (top) to highest (bottom) degree of association with genes from the data set by the *P*-value of a right tailed Fishers exact *t*-test. The Benjamini-Hochberg test for multiple comparisons determined that these pathways all had –log P value **=** 0.23. **B** The bar chart on the left represents the percentage of genes differentially regulated from the data set, which map to an enriched GO term of interest classified as a biological process. The most significant terms from the analysis were not displayed as these were too general and not informative in terms of specific function. The GO terms were ranked from lowest to highest degree of association with genes from our data set, by the *P*-value calculated using the Benjamini-Yuketeli test for multiple comparisons (top to bottom in graph on right).

The top ten canonical pathways generated in IPA and significant GO terms indicate a trend toward directional cell growth and extracellular signalling. In particular, the three most significantly associated IPA canonical pathways are axonal guidance (Additional file 4: Figure S3), Ephrin A and Rho GTPase signalling, which are associated with cell attachment and cytoskeletal rearrangement (Figure 4A). The IL-6 signalling pathway (Additional file 5: Figure S4), associated with inflammation and acute phase reaction, also contains a number of genes which were activated in large follicles including *IL6R, JNK, PIK3R* and *TSG6* (or *TNFAIP6*, already mentioned). The GO terms enriched for the large to small follicle comparison are also connected with inflammation signalling and cell rearrangement (Figure 4B).

**Figure 5:**
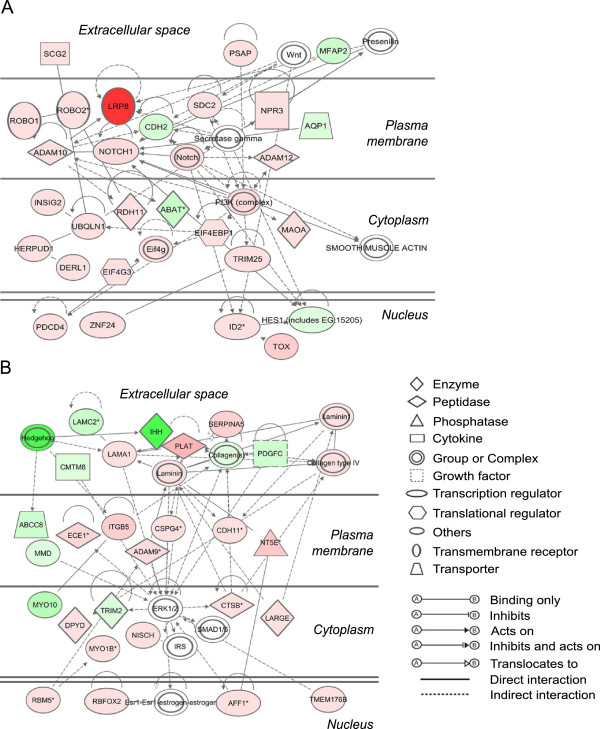
**The two top-ranked networks (A, B) determined for molecules mapped to the IPA database from a data set containing genes differentially regulated between small and large follicles.** Interactions between molecules are shown as explained in the legend, with focus molecule symbols highlighted in color, based on up (red) or down (green) regulation in large follicles and of increasing intensity with degree of fold change.

The two top networks generated by IPA based on the dataset above are shown in Figure 5. The network in Figure 5A shows an emphasis on cytoplasmic membrane receptor signalling centred around Notch and the ADAM protease genes and axonal guidance through the ROBO genes and *LRP8*. There is also considerable connectivity associated with *PI3K* which exerts direct effects on the cytoskeleton and indirectly protein translation via *EI4EBP1*. The other network (Figure 5B) indicates significant interaction with extracellular matrix by *LAMA1*, *LAMC2* and *COL4A1* which appear to mainly signal through the cell surface components *ITGB5*, *CSPG4* and *CDH11* to ERK pathways. This extracellular matrix pathway is probably that associated with focimatrix production that develops as follicles enlarge from 5 to 10 mm in diameter [9,27].

### Genes activated in large versus small follicles

#### TGF-β signalling

It is well known that TGF-β signalling plays an important role in follicular development, as reviewed by Knight and Glister in 2006 [28] and more recently by Myers and Pangas in 2010 [29]. In our study, three members of the TGF-β superfamily, *INHBA* which helps drive androgen production from the theca [30] and inhibits production of FSH by the pituitary [31], and the bone morphogenetic protein receptor genes *BMPR1A* and *BMPR2*, were up regulated in large follicles (Table 1). The BMP receptor type II binds GDF-9 and BMP-15, two critical growth factors for granulosa cells which are secreted by the oocyte at antral stages [32]. The activation of these genes probably contributes to follicle growth during the latter antral stages when androgen production is increased and combines with LH to maintain high oestradiol levels following the reduction in circulating levels of FSH when a dominant follicle emerges.

#### Immune/Inflammation signalling

The immunoregulatory receptor genes*, IL4R IL6R* and *IL20RA* and the thrombin and thrombin-like receptors *F2R* and *F2RL1*were also identified among the list of genes activated in large follicles (Table 1)*.* Bovine granulosa cells have been shown to be capable of initiating an inflammatory response to lipopolysaccharide with increased expression of IL-6 and IL-8 [33]. Additionally, IL-6 and its receptor have been studied in relation to cumulus-oocyte complex development, where they are known to play an active role in expansion and ovulation [34]. The expression of another inflammatory cytokine IL-4 and its receptor have been shown to increase in the rat preovulatory follicle [35]. Interestingly, thrombin receptor RNA expression has previously been reported to be lower in larger follicles than small [36] as opposed to our study, though the health of the follicles was unclear in the other study. These inflammatory pathways identified as significant in our analysis further confirm that significant signalling through these pathways occurs in the later stages of bovine antral follicle development.

#### Axonal guidance

An interesting subset of the signalling genes active in large follicles is concerned with directional cell growth and cellular processes, mainly through the SLIT (*SLITRK1*)/ roundabout or ROBO (*ROBO1*, *ROBO2*) and semaphorin (*SEMA6A*)/plexin (*PLXNB2*, *PLEXNC1*) pathways (Table 2 and Figure 5A). *ROBO1* and *ROBO2*, and *SLITRK2* are part of the SLIT-ROBO pathway, which acts as an important repulsive cellular guidance mechanism to control vascular and mesenchymal tissue development [37]. Whilst follicles do not have a branching structure, during their growth they are expanding within a stromal tissue, as branching ducts are required to do, suggesting that the semaphorin (*SEMA6A*)/plexin (*PLXNB2*, *PLEXNC1*) pathway is important in the process of follicle expansion. Another up-regulated gene *NOTCH1*, can similarly affect cell polarity and tissue structure [38].

In fact, these molecules are known to be present in follicle development in the fetal ovary [39] and adult ovarian follicle [40,41]. LRP8, an endocytosis and cholesterol transport participant, was previously found to be more highly expressed in large versus small antral follicles [42] and in the dominant follicle compared with the subordinate and preovulatory follicles [43]. LRP8 is also crucial for binding ephrins, which are involved with directed growth and cell migration [44]. Ephrin receptors, including A4 and their corresponding ligands, have been demonstrated in human luteinised granulosa cells [45]; but not at earlier stages. The activation of these genes and subsequent axonal guidance pathways identified in our arrays indicate the importance of polarity switching and cell rearrangement as the follicle prepares for ovulation and luteinisation of granulosa cells. Ovulation requires that the follicle and cumulus expand and the oocyte migrate to the point of release facing the ovarian surface, which necessitates coordinated signalling between mural and cumulus granulosa and the oocyte.

#### Protein trafficking

Molecules involved in protein trafficking constitute another important group within our up-regulated data set (Table 1), and some of these participate in cell signalling pathways through the pleckstrin homology domain binding proteins *PLEKHA2, PLEKHB2, PLEKHG1* and *PLEKHH3. PLEKHA2* is a participant in the phosphoinositidyl-3-phosphate kinase (PI3K) signalling pathway which is sensitive to superoxide production [46], possibly as a by-product of steroidogenesis.

#### Transcription factors

As the follicle enlarges the granulosa cells mature and we would expect major changes in the types of molecular pathways which are active in the granulosa cells. This is reflected in the high number of transcriptional regulators of developmental processes encoded by genes like *FOXP2, CREB3L2, JARID2, CITED1* and *CITED2* which are switched on in large follicles (Table 1). The cAMP-responsive element binding protein (CBP) p300 interacting transcriptional modulator *CITED1*, has been shown to be activated by FSH treatment of *in vitro* matured granulosa cells [47], and *CITED2* encodes a factor which competes with hypoxic inducible factor (HIF1α) for CBPp300 [48], and is important for embryonic development of neural tissue [49].

#### Cell growth

Many of the genes identified in this study encode proteins responsible for growth and metabolism. Several of these are known to be involved in follicular development and confirm previous studies, such as IGFBP-4 and-6, which were found to be up regulated in granulosa cells isolated from large follicles [50] (Table 1). Other genes such as chordin-like 1, a BMP-4 antagonist [51], have not previously been associated with follicular development.

#### Intercellular and matrix adhesion

A number of molecules which create intercellular interactions and/or bind extracellular matrix are also encoded by genes which are listed in Table 1. Nine extracellular matrix genes were up regulated in large follicles, and encode proteins including collagen types 4α1 (*COL4A1*) and 16α1 (*COL16A1*), and laminin α1 (*LAMA1*), as well as the proteoglycans, versican (*VCAN*), *CSPG4* and syndecan 2 (*SCD2*) (Figure 5A). This group also contains the most highly expressed gene from the entire list, *TNFAIP6,* almost 280-fold higher expressed in large follicles (Table 2 and Additional file 5: Figure S3). The up-regulated molecules which bind matrix or stabilise intercellular attachment, are represented by the tetraspanins 2 and 9 (*TSPAN2*, *TSPAN9*), the Rho GTPase activating proteins-17 and −18 (*ARHGAP17*, *ARHGAP18*), and the well-known cell surface antigens, integrin β5 (*ITGB5*) and VCAM1, amongst others. Integrin β5 is expressed in mature follicles in the mouse [52] and it is known that integrins bind extracellular matrix and can mediate cell migration, replication or apoptosis [53]. *VCAM1* expression has not previously been associated with granulosa cells in follicle development. It is generally expressed in endothelial cells but can be expressed in other epithelia and promote adhesion of circulating inflammatory cells [54], and thus may also participate in the ovulatory process.

#### Proteolysis and inhibition

There are 13 up-regulated transcripts that encode enzymes which collectively encompass a broad range of proteolytic activities (Table 2) in large follicles. Two highly-expressed transcripts are encoded by the serine protease 23 (*PRSS23*) and tissue plasminogen activator (*PLAT*) genes. This group includes several members of the ADAM family of metalloproteases: *ADAM9*, *ADAM10*, *ADAM12* and *ADAMTS4*. Three well known protease inhibitor genes, *TIMP1, TIMP2* and *SERPINA5*, are also abundantly expressed. Although it is known that ADAMTS1 plays a role in matrix remodelling and is important for ovulation in the mouse [55], horse [56] and human [57] and ADAM8 is regulated by progesterone and luteinising hormone [58], there is little evidence to date concerning the function of ADAM metalloproteases 9, 10 and 12 in the ovarian follicle. These three proteases together are capable of degrading fibronectin and collagen IV, and shed Fas and kit ligand from epithelial cells *in vitro*[59] and thus may regulate the breakdown of matrix and differentiation of granulosa cells prior to ovulation. The inhibitors of matrix metalloproteases, TIMP 1 and 2 are also critical players in the breakdown of matrix close to the time of ovulation [60,61] and can promote progesterone synthesis. An important feature of future studies will be to comprehensively map the spatio-temporal expression of these proteins in the extracellular matrix, and determine the biological effect of their accumulation.

### Genes activated in small versus large follicles

Table 3 shows several important cytokine and receptor genes which have lower expression in large follicles including *KIT*, *PDGFC* (Figure 5B)*, FGFR2, F2RL2, IL33, IL20RA*, and *ANGPT2*. Other interesting highly down regulated genes of various functions include the developmental genes: *MEST* (the most down regulated in large follicles, 28-fold), *IHH* (also Figure 5B) and *MYC*, and also *JAKMIP1*, which participates in cell polarisation.

Two of the genes mentioned before, *KIT*[62,63] and *AMH*[64], are associated with follicle survival and maturation. The imprinted gene *MEST* which is mesodermally expressed in early embryos [65], is also strongly up regulated in small follicles. This developmental gene has been shown to be highly expressed in oocytes compared with cumulus cells [66], but not necessarily throughout the membrana granulosa. *IHH,* one of the hedgehog-signalling family genes found here to be up regulated in small follicles, has been shown to be necessary for proper egg chamber formation in Drosophila [67], and is hormonally regulated and associated with co-maturation of the theca interna in the mammalian ovary [68]. Both IHH and MEST may be necessary for the maintenance of an immature granulosa cell phenotype in small follicles. Interestingly, a related hedgehog family member Sonic Hedgehog Homolog (Shh) has also been reported to be regulated by heparan sulphate proteoglycan binding [69]. These molecules exist in abundance within antral follicles in the form of syndecan and glypican (cumulus cells) [70] and perlecan (between mural granulosa cells) [27], and it is possible that they may play a role at this stage of follicle development.

### Upstream regulator analyses

IPA Upstream Regulator analysis was used to identify upstream transcriptional regulators and the results are shown in Table 4. The validity and usefulness of such analyses is shown by the identification of known important pathways or molecules affecting follicle growth or granulosa cell function such as the gonadotrophin/protein kinase pathways (with identified upstream regulators including chorionic gonadotrophin, follicle-stimulating hormone, forskolin, 8-bromo cAMP, bucladesine which is a cell permeable cAMP analogue, epidermal growth factor pathway (ERBB2), renin angiotensin system (lorsartan which is an inhibitor of the angiotensin Type II receptor), oestradiol (tamoxifen;), leptin (LEPR), inhibin (INHBA), GATA transcription factors (GATA6), , VEGF, retinoid action (AGN194294 which is an RXR ligand), lipid metabolism (APOE,) and the aryl hydrocarbon (AH) receptor (tetrachlorodibenzodioxin).

**Table 4:** **Upstream regulators predicted to be activated or inhibited in large follicles compared with small follicles, using the 3-fold differentially-regulated data set with FDR *P <*** **0.05, on the basis of known interactions compiled in the IPA Upstream Regulator analysis**

**Upstream regulator**	**Molecule type**	*Activation z-score	** *P- * **value of overlap**	**Target genes in the data set**
**Predicted activated molecules/genes**				
Tacrolimus	Chemical drug	3.302	2.1×10^-2^	*ABLIM1, ACTA1, CDK13, COL1A1, CYBB, FOS, ID3, IL33, MYC, PDCD4, PTGS2*
*STAT4*	Transcription regulator	3.300	4.8×10^-2^	*AKAP8L, ARFGAP3, ERRFI1, GLG1, IER3, MGARP, PYGL, RCN3, RNF128, SF3B1, WHSC1L1*
Chorionic gonadotrophin	Hormone	3.224	2.4×10^-9^	*ABCB1, AKR1C3, CDH2, CLU, CYP11A1, CYP19A1, F2RL1, HSD3B2, IER3, IGFBP4, IL33, IL4R, INHBA, ITGB5, LGALS3BP, LHCGR, MTPN, NPR3, NR5A2, NRP1, PFKFB3, PGR, PLAT, PPAP2B, PTGFR, PTGS2, SFRP4, STAR, TIMP1, TNFAIP6, VCAN*
*XBP1*	Transcription regulator	2.887	1.5×10^-3^	*APBB2, APOA1, ARFGAP3, COPZ1, DERL1, EDEM2, GOLGA4, HERPUD1, HM13, HMOX1, MYC, RCN3, RRT1, SEC63, VCAM1*
*FSH*	Hormone	2.759	1.8×10^-4^	*ACTA1, BCL2L2, BMPR1A, BMPR2, CDH2, CITED1, CYP11A1, CYP19A1, FOS, GRK5, HSD3B2, IGFBP4, INHBA, ITGB5, LHCGR, MAPK6, MYC, NOL3, PGR, PLAT, PTGS2, RPRM, STAR, TIMP1, TIMP2, TNFAIP6, TOB1*
*FOXO*3	Transcription regulator	2.613	7.4×10^-2^	*EIF4EBP1, FGFR2, GABARAPL1, GADD45B, IER3, MYC, SLC40A1, TXNIP*
AGN194204	Chemical drug	2.550	2.4×10^-3^	*ANAPC5, BZW2, CLIC4, FDFT1, IL4R, KLF6, MAN1A1, MYC, PDCD4, RCAN3, STIM1, STRA6, TIMP1*
Forskolin	Chemical toxicant	2.444	3.4×10^-5^	*ACTA1, APOA1, ATP6V1A, CARTPT, CDH2, CLU, COL1A1, CYP11A1, CYP19A1, FOS, GRK5, HMOX1, HSD3B2, ID2, ID3, IGFBP6, INHBA, ITGB5, LARGE, LHCGR, LTF, MYC, NOL3, NT5E, PGR, PLAT, PTGS2, PTHLH, RAB7A, RPRM, SCG2, STAR, TIMP1, TNFAIP6, TXNIP, VCAN*
*INHBA*	Growth factor	2.389	3.2×10^-2^	*CPNE8, CYP11A1, CYP19A1, DTNA, INHBA, LHCGR, PRPF38B, STAR, TIMP1*
*GATA6*	Transcription regulator	2.377	5.6×10^-3^	*BMPR2, CYP11A1, CYP19A1, HSD3B2, LHCGR, STAR*
8-bromo-cAMP	Chemical reagent	2.287	2.1×10^-4^	*APOA1, CLU, CYP11A1, CYP19A1, FOS, HSD3B2, LHCGR, MYC, PGR, PLAT, PTGFR, PTGS2, STAR, TIMP1, TIMP2*
Bucladesine	Chemical toxicant	2.166	1.0×10^-4^	*CDH2, CLU, CYP11A1, CYP19A1, ENPP1, ERRFI1, F3, FOS, GADD45B, HMOX1, HSD3B2, IGFBP6, KIT, MYC, PTGS2, QSOX1, RGN, SCG2, STAR, TIMP1, TIMP2*
Vegf	Cytokine	2.008	2.6×10^-4^	*ABCB1, ADAM10, ANGPT2, ANGPTL2, BMP2K, F3, GRK5, HES1, HMOX1, IGFBP4, INHBA, LPHN2, LRP8, MYC, NOTCH1, NR5A2, NRP1, PPAP2B, PTGS2, PTHLH, TIMP1, TRIB2, VCAM1*
**Predicted inhibited molecules/genes**				
LEPR	Transmembrane receptor	−2.000	7.2×10^-2^	*APOA1, APOA2, CARTPT, COL1A1, COL4A1, FOS, TIMP1*
Losartan potassium	Chemical drug	−2.035	6.4×10^-6^	*AQP1, COL1A1, CYBB, F3, FOS, GRK5, ITGB5, PTGS2, PTHLH, STAR, TIMP1, VCAM1*
APOE	Transporter	−2.035	2.5×10^-5^	*APOA1, CLU, COL1A1, CTSB, CYBB, F2R, F2RL1, F3, GPR77, GPX3, HMOX1, IGFBP6, LRP8, MYO1B, NPNT, PPAP2B, PTGS2, TIMP1, VCAM1*
Tetrachlorodi-benzodioxin	Chemical toxicant	−2.038	7.2×10^-2^	*ABCB1, CYP11A1, CYP19A1, FOS, HES1, HMOX1, INSIG2, LHCGR, MYC, MYO10, PTGFR, PTGS2, PTPN13, SLC40A1, SPOCK2, STAR*
NR0B1	Ligand-dependent nuclear receptor	−2.092	2.0×10^-4^	*CYP11A1, CYP19A1, HSD3B2, NR5A2, STAR*
Tamoxifen	Chemical drug	−2.241	4.5×10^-3^	*CDH11, CLU, EPHX1, F2R, FHL2, FOS, HES1, IER3, IGFBP4, MYC, PGR, PTGS2, UGCG*
MGEA5	Enzyme	−2.500	1.9×10^-3^	*ABLIM1, ACSS2, CMTM8, CREB3L2, FDFT1, FERMT2, IGFBP4, IL20RA, IL6R, ITGB5, LPHN2, MYO10, PFKM, PPAP2B, TIMP1, TIMP2*
ERBB2	Kinase	−3.304		*ACTA1, ADAM12, ANGPTL2, ATP6V1A, BEX2, CDH11, CHCHD10, CHST10, CLU, COL1A1, COL4A1, CUL1, CUL3, DERL1, EIF4EBP1, F2R, FOS, GPX3, HES1, ID2, IGFBP4, IGFBP6, KIT, LAMC2, MAN1A1, MAOA, MFAP2, MYC, MYO10, NDRG4, NEDD9, NOTCH1, NPNT, NRP1, PDCD4, PDLIM4, PFKFB3, PLAT, PTGS2, TGIF1, VCAN*

*The bias-corrected z-score is used to infer the activation states of transcriptional regulators. It is calculated from the proportions of genes which are differentially regulated in an expected direction based on the known interactions between the regulator and the genes present in the Ingenuity database. Those genes with a z-score greater or less than two are considered to be either activated or inhibited respectively.

**The *P* value of overlap is the calculated statistical significance of overlap between genes from the dataset and genes that are known to be regulated by the upstream regulator using Fisher’s exact test.

**Figure 6:**
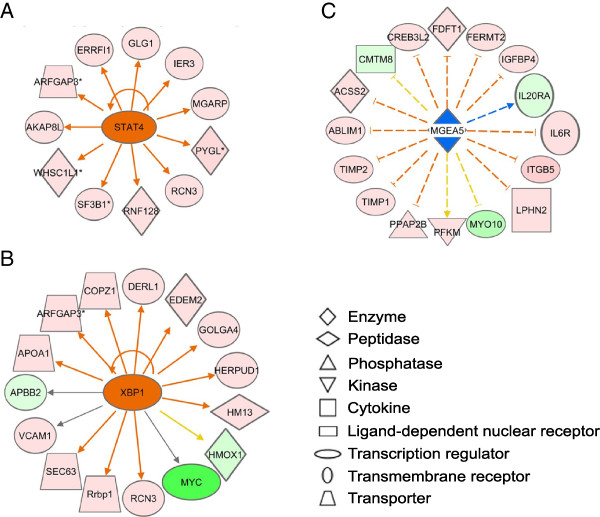
**Two upstream regulators of interest, *STAT4 *(A) and *XBP1 *(B), which are predicted to be activated and one regulator, *MGEA5 *(C), predicted to be inhibited in large follicles based on known interactions with genes in our data set by IPA.** Interactions between molecules are shown as explained in the legend, with focus molecule symbols highlighted in color, based on up (red) or down (green) regulation in large follicles and of increasing intensity with degree of fold change. The suggested action of the central gene is indicated as up-(red) or down-(blue) regulating with the degree of confidence increasing with color intensity. Arrowheads at the end of interactions indicate activation, whereas bars indicate inhibitory effects. The unbroken arrows and the dashed arrows represent direct and indirect interactions respectively, between the genes and the upstream regulators.

Two molecules which have not been well studied in relation to follicular development appear to significantly alter transcription in large follicles: *XBP1* and *STAT4* (Figure 6)*.* XBP1 is cleaved to an activated form under conditions of endoplasmic reticulum stress and subsequently stimulates the expression of a number of chaperones resulting in removal of misfolded proteins and targets them for degradation [71]. It is predicted to be up regulated, and it is possible that radical oxygen species generated by the steroidogenic process may contribute to the misfolding of proteins [72]. STAT4 is also predicted to be up regulated in large follicles, and is a mediator of the interleukin −12 immune response [73,74], and further supports the involvement of inflammatory processes detected within the follicle at this time as indicated by the IPA and GO enrichment analyses.

A new pathway or molecule identified by IPA Upstream Regulator analyses is *MGEA5* (meningioma expressed antigen 5) which was down regulated in large follicles (Figure 6). There is a diverse set of about 600 proteins known to be post-translationally modified by the addition of O-linked N-acetylglucosamine (O-GlcNAc) to their serine and threonine residues by the action of the enzyme O-GlcNAc transferase (OGT/Sxc) [75]. *MGEA5* encodes beta-*N*-acetylglucosaminidase (O-GlcNAcase), whose catalytic activity removes O-GlcNAc from serine and threonine residues in proteins [75]. This cycling of O-GlcNAc to post-translationally modify proteins can therefore regulate the activity of these proteins. O-linked glycosylation has been observed in bovine cumulus cells and linked to the availability of nutrients for the fuel-sensing hexosamine biosynthetic pathway [76,77]. The hexosamine biosynthetic pathway is sensitive to the levels of lipid, glucose and amine which together supply components of O-GlcNAc. Flux in nutrients thereby modulates protein activity by flux in O-linked glycosylation of proteins. Down regulation of *MGEA5* in large follicles suggests that in small follicles there is decreased O-linked glycosylation of proteins and indeed increased O-linked glycosylation of proteins has been observed to be negative for the success of oocyte maturation [76,77].

## Conclusions

In conclusion, substantial changes occur in gene expression in granulosa cells as follicles enlarge from small to large antral sizes. Gene expression becomes less variable, and the processes of axonal guidance, immune signalling and cell rearrangement were most affected in large follicles. Some important networks were associated with: (A) Notch, *SLIT/ROBO* and *PI3K* signalling, and (B) *ITGB5* and extracellular matrix signalling through extracellular signal related kinases (ERKs). Upstream regulator genes which were predicted to be active in large follicles include *STAT4* and *XBP1*, whereas *MGEA5* was predicted to be inhibited. The latter encodes an enzyme that modifies the activity of many target proteins, including those involved in energy sensing, by removal of N-acetylglucosamine from serine and threonine residues. By comparison, developmental processes such as those stimulated by *KIT*, *IHH* and *MEST* were most active in small follicles.

## Methods

For these experiments bovine ovaries were collected as pairs at a local abattoir in South Australia from non-pregnant *Bos taurus* cows, within 20 min of slaughter and transported to the laboratory on ice. Ovary pairs were macroscopically examined for the presence of a corpus luteum to exclude ovaries from non-cycling cows, and large cystic follicles were discarded. Both small (≤ 5 mm in diameter, n = 10) and large (> 12 mm, n = 4) follicles were selected randomly from different animals. The follicles were dissected from each ovary and the diameter measured with the aid of an ocular micrometer. A portion of each follicle, approximately 100 mm^3^, was removed and fixed in 2.5% glutaraldehyde in 0.1 M phosphate buffer (pH 7.25) for subsequent classification of health or atresia, and granulosa cells were collected from the remaining follicle wall. Only healthy follicles were analysed in this study.

### Histological classification of follicles

Following fixation overnight, the portions of each ovary were rinsed several times with buffer and post-fixed in 2% (v/v) aqueous osmium tetroxide for 1 h at 4°C, as described previously [78]. For light microscopic examination of all follicles, 1 μm-thick epoxy sections were cut using glass knives and a Richert-Jung Ultracut E ultramicrotome (Leica Microsystems Pty. Ltd., VIC, Australia), stained with 1% (w/v) aqueous methylene blue and examined using an Olympus BX50 microscope (Olympus Australia Pty. Ltd, VIC, Australia). Healthy and atretic follicles were identified as described previously [18,19] and all healthy follicles, both large and small, selected for the current experiments had no dead or dying granulosa cells. The small follicle phenotype was sub-classified into two types, rounded or columnar, based on the shape of the basally-situated granulosa cells [79,80].

### Isolation of granulosa cells

Following removal of a portion of tissue for microscopic examination, each follicle was transferred to a 35 mm Petri dish containing 1.0 ml Hank’s balanced-salt solution (HBSS) without calcium or magnesium. The granulosa cell layer was removed by gentle rubbing with a glass Pasteur pipette, previously modified by heat sealing the tip into a rounded smooth surface. The HBSS containing the granulosa cells were centrifuged at 500 × g for 7 min at 4°C, the medium was removed by aspiration and the cells washed twice in phosphate-buffered saline. Finally the cells were resuspended in RNA*later* (Ambion, Austin, TX, USA), and stored at −20°C until required.

### RNA isolation

Total RNA was extracted from the granulosa cells of 10 small and 4 large healthy follicles using RNeasy mini kits (Qiagen). The concentration of the RNA was determined by spectrophotometric measurement at 260 nm. For each granulosa cell preparation, 5 μg of RNA was treated with DNA-free (Ambion) according to the manufacturer’s instructions.

### Real time RT-PCR

Synthesis of cDNA and quantitative Reverse Transcriptase Polymerase Chain Reaction (RT-PCR) using plasmid standards were performed as previously [81] and briefly described here. Total RNA (500 ng) was reverse transcribed with SuperScriptIII (Life Technologies, Carlsbad, Ca, USA) using random hexamer primers (Geneworks, Thebarton, SA, Australia) according to the manufacturer’s instructions. The program Primer Express was used to design primers to the bovine sequences of ribosomal *18S, CYP17A1* and *CYP19A1* (Table 5). An ABI Prism 7000 Sequence Detection System (Applied Biosystems, CA, USA) was used for real time RT-PCR detection with SYBR Green (Eppendorf, Hamburg, Germany) and 10 pmoles of forward and reverse primers in a 20 μl reaction. The amplification conditions are described in Table 5. Plasmid standards were generated by cloning amplified products into pCR2.1-TOPO vector (Life Technologies), then transformed into *E. coli* XL1 Blue (Agilent Technologies), extracted and purified. These standards were quantitated by Absorbance at 260 nm and serially diluted over 3 logs then amplified together with the diluted sample cDNA in the real time reaction to determine quantities of RNA expressed as fg/ng 18S ribosomal RNA.

**Table 5:** Primers and conditions used for quantitative RT-PCR

**Target**	**Primer sequences 5′-3′**	**Genbank accession number**	**PCR reaction and conditions**
*CYP19A1*	Forward ggctatgtggacgtgttgacc	NM_174305	2 min 50C, 10 min 95C, 40 × cycles of 15 s 95C and 60 s 60C
	Reverse tgagaaggagagcttgccatg		
*CYP17A1*	Forward accatcagagaagtgctccgaa	NM_174304	2 min 50C, 10 min 95C, 40 × cycles of 15 s 95C and 60 s 60C
	Reverse ccacaacgtctgtgcctttgt		
*18S*	Forward agaaacggctaccacatccaa	DQ2224	2 min 50C, 10 min 95C, 40 × cycles of 15 s 95C and 60 s 60C
	Reverse cctgtattgttatttttcgt		

### Microarray profiling

Following confirmation of the quality of the RNA and cDNA synthesis, hybridisations to GeneChip Bovine Genome Arrays (Affymetrix, CA, USA) and scanning were performed according to Affymetrix protocols at the Australian Genome Research Facility (Walter & Eliza Hall Institute of Medical Research, Parkville, VIC, Australia) as previously [82] and briefly described below. All samples were analysed together using the same batch of arrays. In brief, the starting amount of total RNA for each probe preparation varied between 2 to 5 μg. First-strand cDNA synthesis was performed using a T7-linked oligo-dT primer, followed by second strand synthesis. *In vitro* transcription reactions were performed in batches to generate biotinylated cRNA targets, which were subsequently chemically fragmented at 95°C for 35 min. Twenty μg of the fragmented, biotinylated cRNA was hybridised at 45°C for 16 h to Affymetrix GeneChip Bovine Genome Arrays, which contained 24,128 probe sets representing over 23,000 transcripts and variants, including 19,000 UniGene clusters. The arrays were then washed and stained with streptavidin-phycoerythrin (final concentration 10 μg/ml). Signal amplification was achieved by using a biotinylated anti-streptavidin antibody. The array was then scanned according to the manufacturer’s instructions. The scanned images were inspected for the presence of any defect on the array.

### Data normalisation and analyses

To minimise discrepancies due to variables such as sample preparation, hybridisation conditions, staining, or array lot, the raw expression data was normalised using the RMA background correction (Robust Multi-array average [83]) with quantile normalisation, log base 2 transformation and mean probe set summarisation with adjustment for GC content and performed in Partek Genomics Suite Software version 6.5 (Partek Incorporated, St Louis, MO, USA). All samples sent for analysis passed all quality controls during analysis. The arrays were analysed as part of a larger set of CEL files which additionally included samples of granulosa RNA from 5 atretic follicles as discussed elsewhere [82]. For initial statistical analysis, the data were first subjected to Principal Component Analysis (PCA, based on the method of [84]) and hierarchical clustering analysis to compare the gene expression patterns of the arrays in terms of our classification. Hierarchical clustering was performed using the Euclidian algorithm for dissimilarity with average linkage. The expression data were analysed by ANOVA using method of moments estimation [85] with post-hoc FDR test for multiple comparisons. The fold change in expression for each gene was based on the non log-transformed values after correction and normalisation. A differentially-expressed gene data set was imported into IPA and genes mapped against the Ingenuity Knowledge Base for network and pathway analysis. These differentially-expressed genes were further annotated and classified based on the GO consortium annotations from the GO *Bos taurus* database (2010/02/24) [86] using GOEAST [87]). The background for the gene enrichment analyses in IPA and GOEAST was the whole array. Statistical association for mapping of genes to functions and pathways in IPA was conducted using a Fisher’s right tailed *t*-test and similarly ranking of mapping to GO terms in GOEAST was accomplished by the Benjamini-Yuketeli method. Expression data were also exported to Excel and used to generate size-frequency distributions of the coefficient of variation for each probe set for small and large follicles. We also used IPA Upstream Regulator analysis to identify upstream transcriptional regulators by Fisher’s exact *t*-test. The analytical outcome is based upon prior knowledge of expected effects between transcriptional regulators and target genes stored in the Ingenuity Knowledge Base. The microarray CEL files, normalised data and experimental information have been deposited in the GEO database under series record GSE39589.

## Abbreviations

FDR: Benjamini-Hochberg false discovery rate; GO: Gene ontology; GOEAST: Gene ontology enrichment analysis software toolkit; GEO: Gene expression omnibus; HBBS: Hank’s balanced-salt solution; IPA: Ingenuity pathway analysis; O-GlcNAc: O-linked N-acetylglucosamine; PCA: Principal component analysis; RMA: Robust multi-array average; RT-PCR: Reverse transcriptase polymerase chain reaction.

## Competing interests

No competing interest, financial or otherwise, are declared by the authors.

## Authors’ contributions

Conceived and designed the experiments: HFI-R, RJR. Performed the experiments: NH, KH, HFI-R, MLH, SEM. Analysed the data: NH, KH, RJR. Contributed reagents/materials/analysis tools: NH, KH, RJR. Wrote the paper: NH, KH, HFI-R, RJR. All authors read and approved the final manuscript.

## Supplementary Material

Additional file 1: Figure S1 Unsupervised hierarchical clustering across all probe sets (n = 24,182) for 14 arrays using the Euclidian dissimilarity algorithm method with average linkage in Partek. The heatmap represents the distribution of normalised signal intensity, grouping by pattern similarity for both probe set and array. The R columns represent the rounded granulosa cells and the C columns represent the columnar granulosa cell arrays. Click here for file

Additional file 2: Figure S2 Plots of coefficients of variation (CV) versus their frequency for granulosa cell cDNA hybridised to Bovine Genome Affymetrix Expression arrays across replicate samples per gene for small (n = 10) and large follicles (n = 4). The 50% most highly expressed genes, representing half of all probe sets (n = 12,064) were used in these analyses. 2 fold and 3 fold represent all probe sets which were 2-fold (n = 1809) or 3-fold (n = 598) differentially regulated between small and large follicles in Partek. Click here for file

Additional file 3: Table S1 The total number of probe sets (758) which were 3-fold differentially regulated with a Benjamini-Hochberg FDR multiple correction of *P <* 0.05 between large and small healthy follicles listed in alphabetical order by gene symbol. Click here for file

Additional file 4: Figure S3 The complete canonical Axonal Guidance Signalling pathway as presented in IPA showing which genes map from the 3-fold differentially-expressed dataset with a Benjamini-Hochberg FDR multiple correction *P <* 0.05 between large and small healthy follicles. Genes which are up regulated in large are indicated in red, and those which are down regulated are green, with the degree of fold difference commensurate with the color intensity. Click here for file

Additional file 5: Figure S4 The complete canonical IL-6 signalling pathway as presented in IPA showing which genes map from the 3-fold differentially-expressed dataset with a Benjamini-Hochberg FDR multiple correction *P <* 0.05 between small and large healthy follicles. Genes which are up-regulated in large are indicated in red, and those which are down-regulated are green, with the degree of fold difference commensurate with the color intensity. Click here for file
