# Supplementary figures and images for: Transcriptome profiling of granulosa cells of bovine ovarian follicles during growth from small to large antral sizes
Source: BMC Genomics. 2014 Jan 14;15:24. doi: 10.1186/1471-2164-15-24 (PMC3898003; doi:10.1186/1471-2164-15-24)

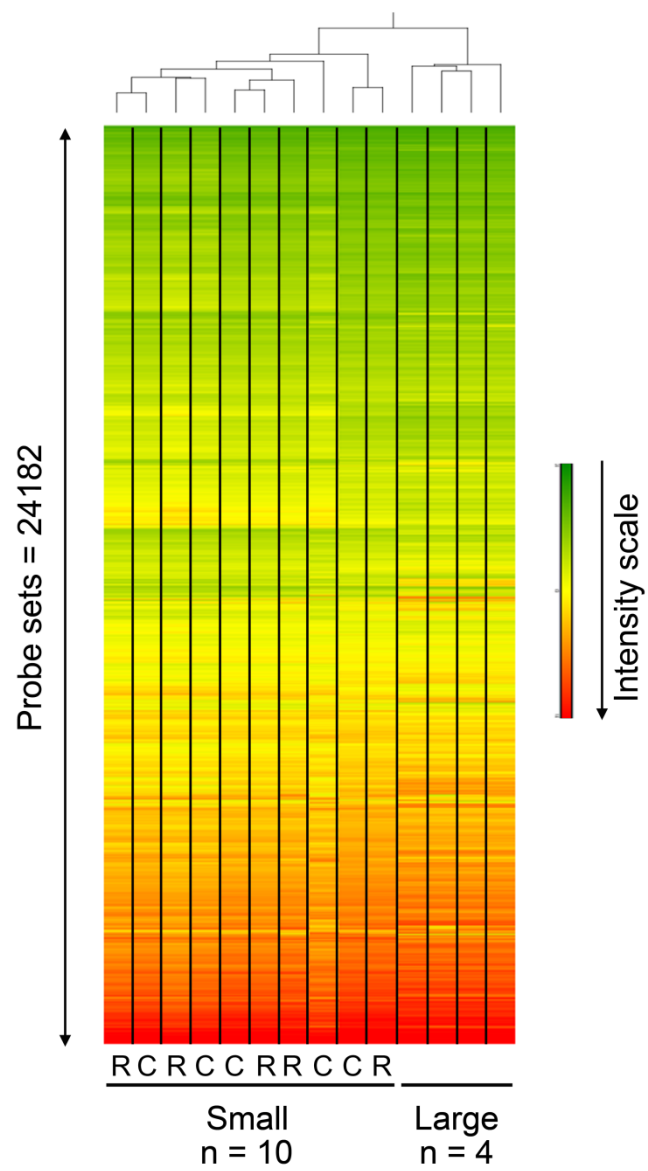

Supplement: Additional file 1: Figure S1 — Unsupervised hierarchical clustering across all probe sets (n = 24,182) for 14 arrays using the Euclidian dissimilarity algorithm method with average linkage in Partek. The heatmap represents the distribution of normalised signal intensity, grouping by pattern similarity for both probe set and array. The R columns represent the rounded granulosa cells and the C columns represent the columnar granulosa cell arrays. [file 1471-2164-15-24-S1.pdf]

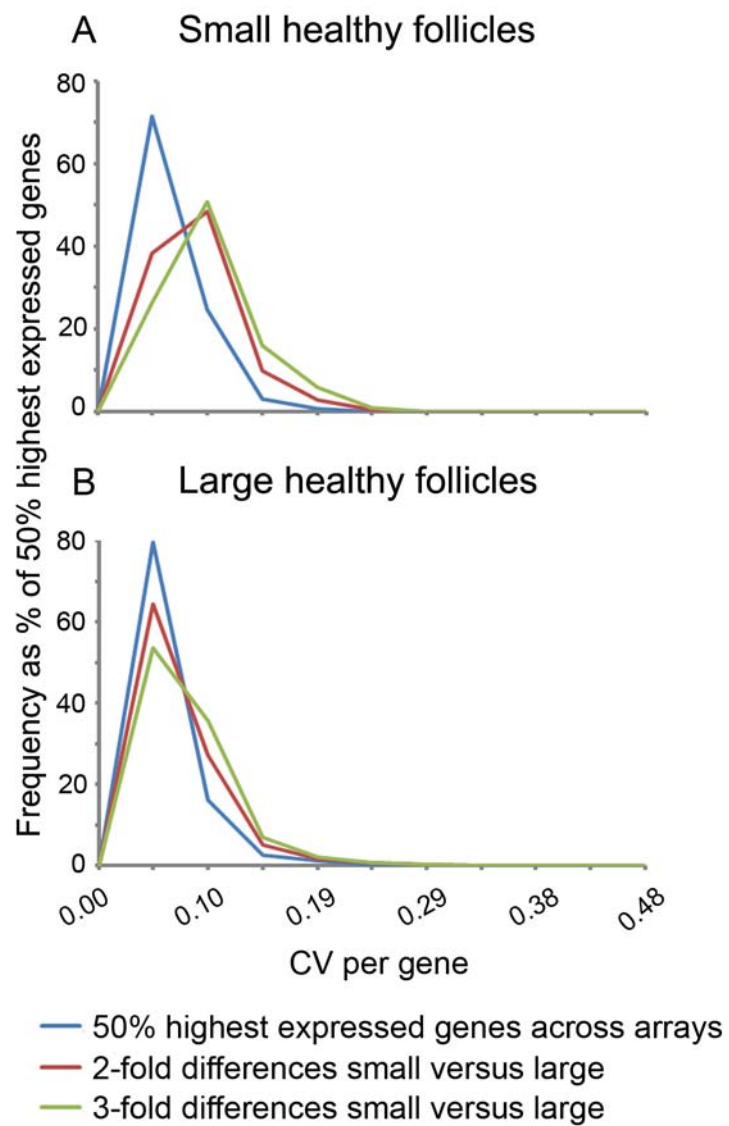

Supplement: Additional file 2: Figure S2 — Plots of coefficients of variation (CV) versus their frequency for granulosa cell cDNA hybridised to Bovine Genome Affymetrix Expression arrays across replicate samples per gene for small (n = 10) and large follicles (n = 4). The 50% most highly expressed genes, representing half of all probe sets (n = 12,064) were used in these analyses. 2 fold and 3 fold represent all probe sets which were 2-fold (n = 1809) or 3-fold (n = 598) differentially regulated between small and large follicles in Partek. [file 1471-2164-15-24-S2.pdf]
