# Supplementary material for: Transcriptome profiling of granulosa cells of bovine ovarian follicles during growth from small to large antral sizes
Source: BMC Genomics. 2014 Jan 14;15:24. doi: 10.1186/1471-2164-15-24 (PMC3898003; doi:10.1186/1471-2164-15-24)
Supplement: Additional file 3: Table S1 — The total number of probe sets (758) which were 3-fold differentially regulated with a Benjamini-Hochberg FDR multiple correction of P < 0.05 between large and small healthy follicles listed in alphabetical order by gene symbol. [file 1471-2164-15-24-S3.pdf]

**Probe set ID**	**Gene Symbol**	**Entrez Gene Name**	**Fold Change**	***P*-value**
Bt.14570.2.S1_at	*ABAT*	4-aminobutyrate aminotransferase	-3.54	7.21E-04
Bt.14570.1.A1_at	*ABAT*	4-aminobutyrate aminotransferase	-5.22	3.84E-03
Bt.15958.2.A1_a_at	*ABCB1*	ATP-binding cassette, sub-family B (MDR/TAP), member 1	5.142	7.19E-08
Bt.1074.1.A1_a_at	*ABCC8*	ATP-binding cassette, sub-family C (CFTR/MRP), member 8	-4.679	1.11E-03
Bt.16041.2.S1_at	*ABLIM1*	actin binding LIM protein 1	3.734	7.23E-03
Bt.401.1.S1_at	*ACBD5*	acyl-CoA binding domain containing 5	3.482	7.14E-07
Bt.28278.1.S1_at	*ACE2*	angiotensin I converting enzyme (peptidyl-dipeptidase A) 2	12.866	1.41E-09
Bt.12640.1.S1_at	*ACSS2*	acyl-CoA synthetase short-chain family member 2	3.478	4.59E-07
AFFX-Bt-actin-3_at	*ACTA1*	actin, alpha 1, skeletal muscle	-3.374	3.56E-03
Bt.4781.1.S1_at	*ADAM10*	ADAM metallopeptidase domain 10	3.656	2.99E-04
Bt.29890.1.S1_at	*ADAM12*	ADAM metallopeptidase domain 12	6.564	3.40E-06

Bt.13118.1.S1_at	*ADAM9*	ADAM metallopeptidase domain 9	8.421	1.50E-08
Bt.734.1.S1_at	*ADAM9*	ADAM metallopeptidase domain 9	4.016	3.43E-07
Bt.12067.1.S1_at	*ADAM9*	ADAM metallopeptidase domain 9	3.237	1.81E-06
Bt.15675.1.S1_at	*ADAMTS4*	ADAM metallopeptidase with thrombospondin type 1 motif, 4	4.657	1.86E-05
Bt.22379.1.S1_at	*ADCK3*	aarF domain containing kinase 3	-3.415	4.36E-04
Bt.3053.1.A1_at	*ADNP*	activity-dependent neuroprotector homeobox	3.679	1.65E-06
Bt.15476.1.A1_at	*AFF1*	AF4/FMR2 family, member 1	5.532	1.22E-06
Bt.12215.1.S1_at	*AFF1*	AF4/FMR2 family, member 1	5.074	1.14E-07
Bt.20733.1.S1_at	*AHCYL2*	adenosylhomocysteinase-like 2	4.395	6.25E-09
Bt.4892.1.A1_at	*AKAP8L*	A kinase (PRKA) anchor protein 8-like	3.642	9.67E-05
Bt.1330.1.S1_at	*AKR1B1*	aldo-keto reductase family 1, member B1 (aldose reductase)	-4.501	4.77E-09
Bt.23094.3.S1_s_at	*AKR1C3*	aldo-keto reductase family 1, member C3 (3-alpha hydroxysteroid dehydrogenase, type II)	-3.059	1.47E-05
Bt.12294.2.A1_at	*AMIGO2*	adhesion molecule with Ig-like domain 2	4.728	9.22E-06

Bt.833.2.S1_at	*ANAPC5*	anaphase promoting complex subunit 5	3.14	6.05E-04
Bt.137.1.S1_at	*ANGPT2*	angiopoietin 2	-11.512	1.06E-03
Bt.21589.1.S1_at	*ANGPTL2*	angiopoietin-like 2	-3.094	2.79E-08
Bt.14043.2.S1_at	*ANKRD10*	ankyrin repeat domain 10	3.107	5.33E-04
Bt.22626.2.S1_at	*ANKRD12*	ankyrin repeat domain 12	3.36	1.58E-06
Bt.21853.1.S1_at	*AP1S2*	adaptor-related protein complex 1, sigma 2 subunit	3.503	1.00E-05
Bt.13163.1.S1_at	*AP2B1*	adaptor-related protein complex 2, beta 1 subunit	6.819	5.14E-06
Bt.10002.1.S1_at	*AP3B2*	adaptor-related protein complex 3, beta 2 subunit	-5.464	4.48E-04
Bt.8933.2.S1_at	*AP3S2*	adaptor-related protein complex 3, sigma 2 subunit	3.556	2.30E-03
Bt.19633.1.A1_at	*APBB2*	amyloid beta (A4) precursor protein-binding, family B, member 2	-3.152	1.18E-08
Bt.6907.1.S1_at	*APC*	adenomatous polyposis coli	3.991	4.61E-07
Bt.1229.1.S1_at	*APOA1*	apolipoprotein A-I	3.187	4.31E-03
Bt.23171.1.S1_at	*APOA2*	apolipoprotein A-II	12.532	3.63E-12

Bt.29460.1.A1_at	*AQP1*	aquaporin 1 (Colton blood group)	-3.179	2.20E-05
Bt.7439.1.S1_at	*ARFGAP3*	ADP-ribosylation factor GTPase activating protein 3	7.554	2.41E-06
Bt.6257.1.S1_at	*ARFGAP3*	ADP-ribosylation factor GTPase activating protein 3	3.747	3.87E-06
Bt.17026.1.S1_at	*ARHGAP17*	Rho GTPase activating protein 17	4.795	8.77E-07
Bt.14056.1.A1_at	*ARHGAP18*	Rho GTPase activating protein 18	20.533	5.29E-12
Bt.3236.1.A1_at	*ARHGEF3*	Rho guanine nucleotide exchange factor (GEF) 3	4.812	3.98E-05
Bt.24927.1.A1_at	*ARHGEF6*	Rac/Cdc42 guanine nucleotide exchange factor (GEF) 6	3.476	2.80E-06
Bt.23192.1.S1_at	*ARPC1B*	actin related protein 2/3 complex, subunit 1B, 41kDa	3.29	2.83E-05
Bt.20559.2.A1_at	*ATP13A3*	ATPase type 13A3	3.63	1.58E-04
Bt.20559.1.S1_at	*ATP13A3*	ATPase type 13A3	3.019	1.98E-04
Bt.4773.2.S1_a_at	*ATP6V1A*	ATPase, H+ transporting, lysosomal 70kDa, V1 subunit A	4.309	4.31E-05
Bt.4773.3.S1_a_at	*ATP6V1A*	ATPase, H+ transporting, lysosomal 70kDa, V1 subunit A	3.064	4.80E-05
Bt.7447.1.A1_at	*B3GALT2*	UDP-Gal:betaGlcNAc beta 1,3-galactosyltransferase, polypeptide 2	6.456	1.94E-07

Bt.7251.1.S1_at	*B3GALT2*	UDP-Gal:betaGlcNAc beta 1,3-galactosyltransferase, polypeptide 2	3.546	1.26E-06
Bt.1225.1.S1_at	*BAI2*	brain-specific angiogenesis inhibitor 2	3.281	1.56E-08
Bt.13974.1.A1_at	*BANK1*	B-cell scaffold protein with ankyrin repeats 1	-3.093	1.39E-05
Bt.1837.1.S1_at	*BCAS3*	breast carcinoma amplified sequence 3	6.022	3.58E-14
Bt.11527.1.A1_at	*BCL2L2*	BCL2-like 2	3.168	1.11E-04
Bt.16678.1.S1_at	*BEX2*	brain expressed X-linked 2	9.006	3.99E-06
Bt.11349.1.A1_at	*BMP2K*	BMP2 inducible kinase	3.79	5.98E-08
Bt.18693.1.S1_at	*BMPR1A*	bone morphogenetic protein receptor, type IA	3.053	1.16E-05
Bt.25715.1.A1_at	*BMPR2*	bone morphogenetic protein receptor, type II (serine/threonine kinase)	3.307	7.61E-07
Bt.27982.1.S1_at	*BMPR2*	bone morphogenetic protein receptor, type II (serine/threonine kinase)	3.123	4.04E-09
Bt.17521.1.S1_at	*BRWD1*	bromodomain and WD repeat domain containing 1	3.258	5.78E-05
Bt.26037.1.A1_at	*BRWD3*	bromodomain and WD repeat domain containing 3	3.234	1.26E-05
Bt.27040.1.A1_at	*BTBD7*	BTB (POZ) domain containing 7	4.315	1.06E-04

Bt.24946.1.A1_at	*Btnl1*	butyrophilin-like 1	-3.064	4.18E-05
Bt.23604.1.S1_at	*BZW1*	basic leucine zipper and W2 domains 1	4.153	3.16E-04
Bt.5495.1.S1_at	*BZW2*	basic leucine zipper and W2 domains 2	3.589	2.25E-04
Bt.5329.2.S1_at	*C10orf57*	chromosome 10 open reading frame 57	5.125	4.70E-08
Bt.5329.1.S1_at	*C10orf57*	chromosome 10 open reading frame 57	3.099	7.14E-07
Bt.10131.1.A1_at	*C16orf52*	chromosome 16 open reading frame 52	3.585	2.14E-05
Bt.8736.2.S1_at	*C1orf9*	chromosome 1 open reading frame 9	3.009	3.90E-05
Bt.26513.1.S1_at	*C2orf40*	chromosome 2 open reading frame 40	-15.011	9.88E-05
Bt.5164.1.S1_at	*CA14*	carbonic anhydrase XIV	-8.847	7.75E-05
Bt.22726.1.A1_at	*CA8*	carbonic anhydrase VIII	3.037	3.34E-03
Bt.27985.1.S1_at	*CANX*	calnexin	3.079	1.19E-04
Bt.16175.1.A1_at	*CAPRIN2*	caprin family member 2	4.485	2.26E-03
Bt.20015.1.S1_at	*CARTPT*	CART prepropeptide	-14.032	4.67E-03

Bt.3946.3.S1_at	*CCDC3*	coiled-coil domain containing 3	-3.197	1.48E-04
Bt.7707.1.S1_at	*CCM2*	cerebral cavernous malformation 2	3.587	1.73E-07
Bt.2696.1.A1_at	*CDH11*	cadherin 11, type 2, OB-cadherin (osteoblast)	4.602	4.11E-02
Bt.2696.2.S1_at	*CDH11*	cadherin 11, type 2, OB-cadherin (osteoblast)	4.519	1.99E-02
Bt.4417.1.S1_at	*CDH2*	cadherin 2, type 1, N-cadherin (neuronal)	-4.043	1.18E-06
Bt.17032.1.A1_at	*CDK13*	cyclin-dependent kinase 13	4.331	6.70E-09
Bt.16843.1.A1_at	*CHCHD10*	coiled-coil-helix-coiled-coil-helix domain containing 10	3.297	6.52E-07
Bt.19645.1.A1_at	*CHD1*	chromodomain helicase DNA binding protein 1	4.693	2.01E-05
Bt.4354.2.S1_at	*CHST10*	carbohydrate sulfotransferase 10	3.084	1.97E-03
Bt.4437.1.S1_at	*CITED1*	Cbp/p300-interacting transactivator, with Glu/Asp-rich carboxy-terminal domain, 1	3.052	8.08E-06
Bt.21607.1.S1_at	*CITED2*	Cbp/p300-interacting transactivator, with Glu/Asp-rich carboxy-terminal domain, 2	3.101	6.91E-04
Bt.17314.1.S1_at	*CLGN*	calmegin	3.164	4.39E-04
Bt.981.1.S1_at	*CLIC4*	chloride intracellular channel 4	3.68	1.03E-06

Bt.4786.1.S1_at	*CLTC*	clathrin, heavy chain (Hc)	3.156	1.84E-04
Bt.12504.1.S1_at	*CLU*	clusterin	-4.894	2.24E-05
Bt.21869.1.S1_at	*CMAHP*	cytidine monophospho-N-acetylneuraminic acid hydroxylase, pseudogene	4.634	8.17E-06
Bt.16243.1.S1_at	*CMTM8*	CKLF-like MARVEL transmembrane domain containing 8	-3.417	1.93E-06
Bt.12697.1.S1_a_at	*COL16A1*	collagen, type XVI, alpha 1	5.955	2.46E-06
Bt.12697.1.S1_at	*COL16A1*	collagen, type XVI, alpha 1	4.323	7.19E-11
Bt.8549.1.S2_at	*COL1A1 (includes EG:1277)*	collagen, type I, alpha 1	-3.003	7.61E-04
Bt.12912.1.S1_at	*COL4A1*	collagen, type IV, alpha 1	6.839	2.82E-08
Bt.12912.1.S2_at	*COL4A1*	collagen, type IV, alpha 1	4.497	1.49E-06
Bt.20999.1.S1_at	*COLEC11*	collectin sub-family member 11	3.51	4.82E-06
Bt.3907.1.S1_at	*COPZ1*	coatomer protein complex, subunit zeta 1	3.252	6.59E-03
Bt.1785.1.A1_at	*CPD*	carboxypeptidase D	11.772	1.69E-07
Bt.19937.2.S1_at	*CPD*	carboxypeptidase D	6.111	6.78E-09

Bt.1785.2.A1_at	*CPD*	carboxypeptidase D	5.053	2.75E-07
Bt.19937.1.S1_at	*CPD*	carboxypeptidase D	4.082	8.51E-09
Bt.18995.1.A1_a_at	*CPEB1*	cytoplasmic polyadenylation element binding protein 1	-3.871	8.24E-05
Bt.20407.2.A1_at	*CPEB4*	cytoplasmic polyadenylation element binding protein 4	5.085	1.80E-09
Bt.25663.1.A1_at	*CPNE8*	copine VIII	3.673	6.43E-07
Bt.8508.1.A1_at	*CREB3L2*	cAMP responsive element binding protein 3-like 2	3.72	6.65E-07
Bt.19146.1.A1_at	*CSDE1*	cold shock domain containing E1, RNA-binding	4.706	3.61E-05
Bt.28247.1.A1_s_at	*CSPG4*	chondroitin sulfate proteoglycan 4	5.53	8.10E-07
Bt.8114.1.A1_at	*CSPG4*	chondroitin sulfate proteoglycan 4	4.668	6.57E-07
Bt.2943.1.A1_at	*CTR9*	Ctr9, Paf1/RNA polymerase II complex component, homolog (S. cerevisiae)	3.869	8.41E-08
Bt.17926.1.A1_at	*CTSB*	cathepsin B	4.445	2.36E-08
Bt.393.1.S1_at	*CTSB*	cathepsin B	3.755	3.32E-08
Bt.393.1.S2_at	*CTSB*	cathepsin B	3.263	2.20E-05

Bt.29888.1.S1_at	*CUL1*	cullin 1	3.001	2.21E-04
Bt.3528.2.S1_at	*CUL3*	cullin 3	3.539	3.19E-04
Bt.18003.1.S1_at	*CUL3*	cullin 3	3.228	2.38E-04
Bt.4558.1.S1_at	*CYBB*	cytochrome b-245, beta polypeptide	18.056	2.24E-14
Bt.22430.1.A1_at	*CYBB*	cytochrome b-245, beta polypeptide	15.856	1.45E-12
Bt.6465.1.S1_at	*CYCS*	cytochrome c, somatic	3.217	2.20E-03
Bt.7190.1.S1_at	*CYP11A1*	cytochrome P450, family 11, subfamily A, polypeptide 1	5.837	3.33E-03
Bt.4447.1.S1_at	*CYP19A1*	cytochrome P450, family 19, subfamily A, polypeptide 1	14.157	3.97E-05
Bt.4447.2.S1_a_at	*CYP19A1*	cytochrome P450, family 19, subfamily A, polypeptide 1	13.19	1.54E-09
Bt.29113.1.A1_at	*CYP2C19*	cytochrome P450, family 2, subfamily C, polypeptide 19	-3.075	8.37E-06
Bt.2726.1.S1_at	*DACT1*	dapper, antagonist of beta-catenin, homolog 1 (Xenopus laevis)	8.43	4.61E-06
Bt.2726.2.S1_at	*DACT1*	dapper, antagonist of beta-catenin, homolog 1 (Xenopus laevis)	7.661	7.77E-07
Bt.3883.1.S1_at	*DDO*	D-aspartate oxidase	-3.585	4.79E-03

Bt.18048.2.S1_at	*DDX26B*	DEAD/H (Asp-Glu-Ala-Asp/His) box polypeptide 26B	9.747	9.83E-08
Bt.18048.1.A1_at	*DDX26B*	DEAD/H (Asp-Glu-Ala-Asp/His) box polypeptide 26B	5.176	2.11E-07
Bt.6783.1.A1_at	*DDX46*	DEAD (Asp-Glu-Ala-Asp) box polypeptide 46	3.353	1.04E-04
Bt.16389.1.S1_at	*DERL1*	derlin 1	4.135	5.10E-07
Bt.6816.1.S1_at	*DICER1*	dicer 1, ribonuclease type III	3.144	5.04E-07
Bt.2506.1.S1_at	*DKK3*	dickkopf 3 homolog (Xenopus laevis)	5.265	1.25E-02
Bt.2424.1.S1_at	*DPYD*	dihydropyrimidine dehydrogenase	3.429	4.87E-05
Bt.5224.1.S1_at	*DPYSL2*	dihydropyrimidinase-like 2	3.787	8.73E-05
Bt.28159.1.S1_at	*DTNA*	dystrobrevin, alpha	8.495	4.68E-08
Bt.4378.2.S1_a_at	*ECE1*	endothelin converting enzyme 1	9.504	2.72E-08
Bt.4378.1.S1_at	*ECE1*	endothelin converting enzyme 1	4.353	3.13E-09
Bt.24750.2.S1_a_at	*ECE1*	endothelin converting enzyme 1	4.196	1.81E-05
Bt.21300.1.S1_at	*EDEM2*	ER degradation enhancer, mannosidase alpha-like 2	3.146	4.31E-07

Bt.3278.1.A1_at	*EFNA5*	ephrin-A5	8.559	3.96E-07
Bt.19309.1.S1_at	*EFNA5*	ephrin-A5	8.536	2.63E-07
Bt.19309.3.A1_at	*EFNA5*	ephrin-A5	8.098	1.63E-06
Bt.21912.1.S1_at	*EIF2C2*	eukaryotic translation initiation factor 2C, 2	3.32	3.37E-05
Bt.29874.1.S1_at	*EIF2C3*	eukaryotic translation initiation factor 2C, 3	3.403	2.37E-06
Bt.5389.1.S1_at	*EIF4EBP1*	eukaryotic translation initiation factor 4E binding protein 1	7.319	4.09E-07
Bt.18267.1.A1_at	*EIF4G3*	eukaryotic translation initiation factor 4 gamma, 3	7.359	2.41E-07
Bt.27284.1.S1_at	*EIF4H*	eukaryotic translation initiation factor 4H	3.486	1.57E-02
Bt.1664.1.A1_at	*EMX2*	empty spiracles homeobox 2	-3.12	2.63E-05
Bt.13899.1.A1_at	*ENPP1*	ectonucleotide pyrophosphatase/phosphodiesterase 1	-4.122	4.61E-04
Bt.8881.1.A1_at	*EPHA1*	EPH receptor A1	-3.151	2.08E-06
Bt.23354.1.S1_at	*EPHX1*	epoxide hydrolase 1, microsomal (xenobiotic)	-3.356	9.32E-05
Bt.23905.1.A1_at	*ERRFI1*	ERBB receptor feedback inhibitor 1	3.554	1.28E-04

Bt.28503.1.A1_at	*EXOC3L4*	exocyst complex component 3-like 4	-3.8	4.77E-03
Bt.20381.2.S1_at	*F2R*	coagulation factor II (thrombin) receptor	8.942	1.65E-11
Bt.10814.1.S1_at	*F2R*	coagulation factor II (thrombin) receptor	5.896	1.16E-06
Bt.20381.1.A1_at	*F2R*	coagulation factor II (thrombin) receptor	5.727	2.62E-08
Bt.28745.1.S1_at	*F2RL1*	coagulation factor II (thrombin) receptor-like 1	5.338	1.22E-04
Bt.24447.2.S1_at	*F2RL2*	coagulation factor II (thrombin) receptor-like 2	-3.077	4.94E-05
Bt.4106.1.S1_at	*F3*	coagulation factor III (thromboplastin, tissue factor)	7.163	3.22E-10
Bt.26767.1.S1_at	*FAF2*	Fas associated factor family member 2	3.181	1.04E-05
Bt.16205.1.A1_at	*FAM114A1*	family with sequence similarity 114, member A1	18.837	8.27E-07
Bt.2177.2.S1_at	*FAM114A1*	family with sequence similarity 114, member A1	5.195	2.42E-04
Bt.15812.1.A1_at	*FAM126B*	family with sequence similarity 126, member B	3.632	1.86E-05
Bt.22149.1.S1_at	*FAM174B*	family with sequence similarity 174, member B	3.367	1.67E-06
Bt.20361.2.A1_at	*FBXL20*	F-box and leucine-rich repeat protein 20	3.911	1.70E-04

Bt.21724.2.S1_a_at	*FDFT1*	farnesyl-diphosphate farnesyltransferase 1	4.081	2.01E-03
Bt.2941.2.S1_a_at	*FERMT2*	fermitin family member 2	4.181	8.72E-06
Bt.5656.1.S1_at	*FEZ1*	fasciculation and elongation protein zeta 1 (zygin I)	-3.817	8.36E-05
Bt.29696.1.A1_at	*FGFR2*	fibroblast growth factor receptor 2	-3.659	6.01E-06
Bt.4451.1.S1_at	*FHL2*	four and a half LIM domains 2	-3.253	1.59E-05
Bt.19999.1.A1_at	*FICD*	FIC domain containing	3.41	6.70E-09
Bt.4882.1.S1_at	*FKBP9*	FK506 binding protein 9, 63 kDa	3.138	9.55E-08
Bt.2899.1.S1_at	*FOS*	FBJ murine osteosarcoma viral oncogene homolog	-4.03	8.88E-04
Bt.2899.1.S2_at	*FOS*	FBJ murine osteosarcoma viral oncogene homolog	-5.512	1.18E-03
Bt.27586.1.A1_a_at	*FOXP2*	forkhead box P2	5.346	7.56E-08
Bt.5369.1.S1_at	*FXYD6*	FXYD domain containing ion transport regulator 6	-3.161	2.65E-08
Bt.3211.1.S1_at	*GABARAPL1*	GABA(A) receptor-associated protein like 1	4.593	1.16E-06
Bt.4371.1.S1_at	*GADD45B*	growth arrest and DNA-damage-inducible, beta	6.827	5.95E-08

Bt.24402.1.S1_at	*GALNT13*	UDP-N-acetyl-alpha-D-galactosamine:polypeptide N-acetylgalactosaminyltransferase 13 (GalNAc-T13)	-3.317	6.59E-07
Bt.24402.2.A1_at	*GALNT13*	UDP-N-acetyl-alpha-D-galactosamine:polypeptide N-acetylgalactosaminyltransferase 13 (GalNAc-T13)	-5.343	4.12E-06
Bt.23250.2.S1_at	*GATM*	glycine amidinotransferase (L-arginine:glycine amidinotransferase)	-11.071	5.98E-06
Bt.2235.1.S1_at	*GDI1*	GDP dissociation inhibitor 1	3.358	9.58E-04
Bt.24597.1.S1_at	*GLG1*	golgi glycoprotein 1	3.335	6.17E-04
Bt.16632.1.A1_at	*Gm16462/Gm8787*	predicted gene 8787	3.034	2.86E-07
Bt.5511.1.S1_at	*GNA11*	guanine nucleotide binding protein (G protein), alpha 11 (Gq class)	3.303	1.04E-05
Bt.18168.1.A1_at	*GOLGA4*	golgin A4	4.645	1.81E-08
Bt.27311.2.S1_at	*GPR173*	G protein-coupled receptor 173	3.001	9.98E-06
Bt.26157.1.A1_at	*GPR77*	G protein-coupled receptor 77	-3.16	6.29E-05
Bt.9893.1.S1_at	*GPR88*	G protein-coupled receptor 88	3.212	1.15E-02
Bt.8998.3.S1_a_at	*GPS2*	G protein pathway suppressor 2	3.085	3.72E-04
Bt.12916.1.S1_at	*GPX3*	glutathione peroxidase 3 (plasma)	3.245	8.76E-03

Bt.4263.1.S1_at	*GRK5*	G protein-coupled receptor kinase 5	5.576	2.99E-05
Bt.178.1.S1_at	*GUCA1A*	guanylate cyclase activator 1A (retina)	-5.124	1.71E-03
Bt.20280.1.S1_at	*GYLTL1B*	glycosyltransferase-like 1B	-3.006	2.17E-02
Bt.26132.1.A1_at	*HECTD1*	HECT domain containing E3 ubiquitin protein ligase 1	3.037	3.58E-05
Bt.7122.1.S1_at	*HELZ*	helicase with zinc finger	3.057	1.80E-03
Bt.20010.1.S1_at	*HERPUD1*	homocysteine-inducible, endoplasmic reticulum stress-inducible, ubiquitin-like domain member 1	3.344	1.82E-04
Bt.18808.1.S1_at	*HES1*	hairy and enhancer of split 1, (Drosophila)	-3.167	3.91E-06
Bt.13535.2.S1_at	*HIATL1*	hippocampus abundant transcript-like 1	3.081	3.64E-05
Bt.3805.1.S1_at	*HLA-A*	major histocompatibility complex, class I, A	-4.436	3.43E-03
Bt.4175.2.S1_at	*HM13*	histocompatibility (minor) 13	4.539	3.05E-04
Bt.9445.1.S1_at	*HMGXB3*	HMG box domain containing 3	3.073	1.03E-05
Bt.4001.1.S1_at	*HMOX1*	heme oxygenase (decycling) 1	-4.088	1.33E-04
Bt.18908.1.S1_at	*HNRPLL*	heterogeneous nuclear ribonucleoprotein L-like	3.865	5.61E-05

Bt.9549.1.S1_at	*HOPX*	HOP homeobox	-13.822	2.37E-05
Bt.4760.1.S1_at	*HSD3B2*	hydroxy-delta-5-steroid dehydrogenase, 3 beta- and steroid delta-isomerase 2	5.955	7.80E-04
Bt.23179.1.S1_at	*HSP90AA1*	heat shock protein 90kDa alpha (cytosolic), class A member 1	6.956	6.17E-04
Bt.21173.1.S1_at	*HUWE1*	HECT, UBA and WWE domain containing 1, E3 ubiquitin protein ligase	3.792	9.77E-05
Bt.2415.1.S1_at	*ID2*	inhibitor of DNA binding 2, dominant negative helix-loop-helix protein	4.473	9.33E-07
Bt.2415.2.S1_at	*ID2*	inhibitor of DNA binding 2, dominant negative helix-loop-helix protein	4.015	6.86E-06
Bt.5230.1.S1_at	*ID3*	inhibitor of DNA binding 3, dominant negative helix-loop-helix protein	4.271	2.68E-05
Bt.3898.1.S1_at	*IDH3A*	isocitrate dehydrogenase 3 (NAD+) alpha	5.464	6.74E-06
Bt.2153.1.S1_at	*IER3*	immediate early response 3	3.277	8.30E-06
Bt.11259.1.S1_at	*Ifi27l1*	interferon, alpha-inducible protein 27 like 1	3.669	2.22E-02
Bt.1548.1.S1_at	*IFI30*	interferon, gamma-inducible protein 30	39.036	5.01E-09
Bt.5237.1.S1_at	*IGFBP4*	insulin-like growth factor binding protein 4	7.33	1.63E-08
Bt.9958.1.S1_at	*IGFBP6*	insulin-like growth factor binding protein 6	3.519	1.39E-03

Bt.22104.1.S1_at	*IHH*	Indian hedgehog	-16.619	6.36E-05
Bt.29391.1.S1_at	*IL20RA*	interleukin 20 receptor, alpha	-3.363	4.19E-05
Bt.22094.1.A1_at	*IL33*	interleukin 33	-3.574	1.03E-04
Bt.24531.1.S1_at	*IL4R*	interleukin 4 receptor	6.188	2.11E-09
Bt.22692.1.A1_at	*IL6R*	interleukin 6 receptor	5.65	6.26E-06
Bt.12760.1.S1_at	*INHBA*	inhibin, beta A	3.814	1.29E-02
Bt.20716.1.S1_at	*INSIG2*	insulin induced gene 2	3.199	9.28E-07
Bt.15806.1.A1_at	*IREB2*	iron-responsive element binding protein 2	3.364	6.82E-05
Bt.5536.1.S1_at	*ITGB5*	integrin, beta 5	11.359	5.47E-12
Bt.19810.1.A1_at	*JAKMIP1*	janus kinase and microtubule interacting protein 1	-20.81	2.84E-07
Bt.7008.1.S1_at	*JARID2*	jumonji, AT rich interactive domain 2	3.177	9.76E-07
Bt.25202.2.S1_at	*KIAA1109*	KIAA1109	3.391	1.38E-05
Bt.25202.3.S1_at	*KIAA1109*	KIAA1109	3.294	1.28E-05

Bt.2361.1.A1_at	*KIAA2026*	KIAA2026	3.214	9.48E-06
Bt.27868.1.S1_at	*KIRREL*	kin of IRRE like (Drosophila)	3.021	1.14E-05
Bt.27675.1.A1_at	*KIT*	v-kit Hardy-Zuckerman 4 feline sarcoma viral oncogene homolog	-4.07	4.22E-05
Bt.26445.1.A1_at	*KIT*	v-kit Hardy-Zuckerman 4 feline sarcoma viral oncogene homolog	-23.094	7.99E-06
Bt.24522.3.S1_a_at	*KLF6*	Kruppel-like factor 6	3.468	1.90E-05
Bt.24380.1.A1_at	*KLHL28*	kelch-like 28 (Drosophila)	5.029	1.00E-09
Bt.26511.2.S1_at	*LAMA1*	laminin, alpha 1	5.78	1.79E-06
Bt.6509.3.S1_a_at	*LAMC2*	laminin, gamma 2	-3.398	7.19E-04
Bt.6509.1.S1_at	*LAMC2*	laminin, gamma 2	-5.085	2.33E-03
Bt.29331.1.S1_at	*LARGE*	like-glycosyltransferase	3.186	1.41E-06
Bt.26572.1.S1_at	*LEPREL1*	leprecan-like 1	4.908	1.17E-04
Bt.6597.2.S1_a_at	*LGALS3BP*	lectin, galactoside-binding, soluble, 3 binding protein	3.547	7.93E-07
Bt.6597.1.S1_at	*LGALS3BP*	lectin, galactoside-binding, soluble, 3 binding protein	3.521	1.20E-05

Bt.37.2.S1_a_at	*LHCGR*	luteinizing hormone/choriogonadotropin receptor	8.759	2.40E-07
Bt.37.1.S1_at	*LHCGR*	luteinizing hormone/choriogonadotropin receptor	7.01	8.92E-07
Bt.15732.1.S1_a_at	*LHFPL2*	lipoma HMGIC fusion partner-like 2	6.755	1.38E-05
Bt.15732.1.S1_at	*LHFPL2*	lipoma HMGIC fusion partner-like 2	3.886	1.42E-04
Bt.3015.2.S1_at	*LHFPL2*	lipoma HMGIC fusion partner-like 2	3.697	6.14E-04
Bt.10730.1.S1_at	*LIMA1*	LIM domain and actin binding 1	8.167	6.11E-07
Bt.17570.2.A1_at	*LINGO2*	leucine rich repeat and Ig domain containing 2	5.01	6.86E-07
Bt.29911.1.S1_at	*LOC388588*	uncharacterized protein LOC388588	3.258	2.02E-09
Bt.23209.1.S1_a_at	*LPHN2*	latrophilin 2	4.727	1.95E-09
Bt.22664.1.A1_at	*LRIG3*	leucine-rich repeats and immunoglobulin-like domains 3	3.675	1.41E-06
Bt.29862.1.A1_at	*LRP8*	low density lipoprotein receptor-related protein 8, apolipoprotein e receptor	53.602	2.43E-12
Bt.24230.1.S1_at	*LRRC1*	leucine rich repeat containing 1	-3.755	2.03E-09
Bt.4886.1.A1_at	*LRRC17*	leucine rich repeat containing 17	-7.101	1.27E-04

Bt.22167.2.A1_at	*LRRC2*	leucine rich repeat containing 2	3.574	2.00E-06
Bt.4802.1.S1_at	*LTF*	lactotransferrin	-3.981	5.75E-04
Bt.4874.2.S1_a_at	*LUC7L3*	LUC7-like 3 (S. cerevisiae)	3.771	2.69E-04
Bt.28686.2.S1_at	*MAL2*	mal, T-cell differentiation protein 2 (gene/pseudogene)	3.797	5.62E-05
Bt.28686.1.S1_at	*MAL2*	mal, T-cell differentiation protein 2 (gene/pseudogene)	3.042	2.36E-04
Bt.24258.2.S1_at	*MAN1A1*	mannosidase, alpha, class 1A, member 1	-3.152	5.37E-03
Bt.24258.1.S1_at	*MAN1A1*	mannosidase, alpha, class 1A, member 1	-4.183	8.64E-04
Bt.9379.1.S1_at	*MAOA*	monoamine oxidase A	3.051	3.03E-05
Bt.23717.1.A1_at	*MAP2K4*	mitogen-activated protein kinase kinase 4	4.428	3.39E-07
Bt.18165.1.A1_at	*MAPK6*	mitogen-activated protein kinase 6	4.84	3.61E-07
Bt.10146.2.S1_at	*MARCH5*	membrane-associated ring finger (C3HC4) 5	3.186	3.90E-05
Bt.26375.1.A1_at	*MARCH6*	membrane-associated ring finger (C3HC4) 6, E3 ubiquitin protein ligase	3.934	3.48E-04
Bt.24594.1.A1_at	*ME3*	malic enzyme 3, NADP(+)-dependent, mitochondrial	26.744	2.12E-14

Bt.24594.2.S1_at	*ME3*	malic enzyme 3, NADP(+)-dependent, mitochondrial	7.333	1.45E-10
Bt.21989.1.A1_at	*MED24*	mediator complex subunit 24	3.099	2.79E-03
Bt.29873.1.S1_at	*MEST*	mesoderm specific transcript homolog (mouse)	-8.899	2.97E-05
Bt.9556.1.S1_at	*MEST*	mesoderm specific transcript homolog (mouse)	-28.678	6.65E-10
Bt.3092.1.S1_at	*MFAP2*	microfibrillar-associated protein 2	-4.599	1.09E-03
Bt.25070.1.S1_at	*MGARP*	mitochondria-localized glutamic acid-rich protein	3.072	2.53E-03
Bt.27718.1.S1_at	*MIA3*	melanoma inhibitory activity family, member 3	5.426	8.28E-08
Bt.25291.1.A1_at	*MIA3*	melanoma inhibitory activity family, member 3	3.632	2.90E-09
Bt.22579.1.S1_at	*MICU1*	mitochondrial calcium uptake 1	3.231	7.51E-06
Bt.27167.1.A1_at	*MLEC*	malectin	3.836	1.05E-04
Bt.24691.1.A1_at	*MLL5*	myeloid/lymphoid or mixed-lineage leukemia 5 (trithorax homolog, Drosophila)	3.17	7.43E-04
Bt.615.1.S1_at	*MMD*	monocyte to macrophage differentiation-associated	-3.077	3.52E-09
Bt.27248.1.A1_at	*MMP16*	matrix metallopeptidase 16 (membrane-inserted)	-3.982	9.97E-09

Bt.25992.1.A1_at	*MPP5*	membrane protein, palmitoylated 5 (MAGUK p55 subfamily member 5)	7.14	1.45E-05
Bt.20102.1.S1_at	*MPV17L2*	MPV17 mitochondrial membrane protein-like 2	3.057	1.94E-05
Bt.24796.1.S1_at	*MRAP*	melanocortin 2 receptor accessory protein	3.252	6.94E-10
Bt.9605.1.S1_at	*MS4A8B*	membrane-spanning 4-domains, subfamily A, member 8B	5.32	1.07E-07
Bt.15556.1.A1_s_at	*MSX1*	msh homeobox 1	-3.094	8.66E-04
Bt.647.1.S1_at	*MTPN*	myotrophin	5.732	4.02E-04
Bt.19087.1.A1_at	*MTR*	5-methyltetrahydrofolate-homocysteine methyltransferase	3.407	2.56E-04
Bt.21164.1.S1_at	*MYC*	v-myc myelocytomatosis viral oncogene homolog (avian)	-16.36	4.11E-10
Bt.17630.1.S1_at	*MYCBP2*	MYC binding protein 2, E3 ubiquitin protein ligase	3.434	6.88E-08
Bt.4506.1.S1_at	*MYO10*	myosin X	-6.752	5.20E-08
Bt.16836.2.A1_at	*MYO1B*	myosin IB	5.07	1.58E-06
Bt.16836.1.S1_at	*MYO1B*	myosin IB	3.058	1.61E-05
Bt.29309.1.A1_at	*MYO1D*	myosin ID	-3.028	2.30E-05

Bt.20356.2.A1_at	*MZB1*	marginal zone B and B1 cell-specific protein	-3.072	1.07E-05
Bt.19574.1.A1_at	*NALCN*	sodium leak channel, non-selective	-6.038	6.82E-05
Bt.27963.1.A1_at	*NCEH1*	neutral cholesterol ester hydrolase 1	3.136	1.27E-03
Bt.25965.1.A1_at	*NCOR1*	nuclear receptor corepressor 1	3.463	9.48E-05
Bt.5683.1.S1_at	*NDRG3*	NDRG family member 3	4.392	4.21E-09
Bt.2881.1.S1_at	*NDRG4*	NDRG family member 4	6.72	1.89E-06
Bt.14239.1.A1_at	*NEDD9*	neural precursor cell expressed, developmentally down-regulated 9	-5.881	1.03E-05
Bt.6219.1.S1_at	*NINJ1*	ninjurin 1	3.169	1.23E-04
Bt.3874.1.S1_at	*NISCH*	nischarin	3.272	1.42E-08
Bt.12285.3.S1_a_at	*NMI*	N-myc (and STAT) interactor	8.42	4.83E-12
Bt.12285.2.S1_a_at	*NMI*	N-myc (and STAT) interactor	6.772	8.47E-09
Bt.1185.1.A1_at	*NOL3*	nucleolar protein 3 (apoptosis repressor with CARD domain)	6.037	2.23E-09
Bt.1185.2.S1_at	*NOL3*	nucleolar protein 3 (apoptosis repressor with CARD domain)	3.966	3.12E-08

Bt.17865.1.A1_at	*NOSTRIN*	nitric oxide synthase trafficker	3.231	4.01E-05
Bt.9948.1.S1_at	*NOTCH1*	notch 1	8.42	1.61E-08
Bt.7393.1.S1_at	*NPNT*	nephronectin	-3.162	7.46E-04
Bt.376.1.S2_at	*NPR3*	natriuretic peptide receptor C/guanylate cyclase C (atrionatriuretic peptide receptor C)	5.236	3.16E-09
Bt.10845.1.S1_at	*NR5A2*	nuclear receptor subfamily 5, group A, member 2	5.509	5.04E-06
Bt.10845.2.A1_at	*NR5A2*	nuclear receptor subfamily 5, group A, member 2	3.378	4.89E-05
Bt.20373.1.S1_at	*NRP1*	neuropilin 1	3.033	7.04E-03
Bt.5515.1.S1_at	*NT5E*	5'-nucleotidase, ecto (CD73)	12.796	9.16E-08
Bt.2367.1.A1_at	*NT5E*	5'-nucleotidase, ecto (CD73)	8.252	1.04E-07
Bt.13911.1.S1_s_at	*NUP210*	nucleoporin 210kDa	-4.718	7.56E-07
Bt.24544.3.S1_at	*NUP85*	nucleoporin 85kDa	3.223	4.45E-03
Bt.4888.1.S1_at	*OBSL1*	obscurin-like 1	5.761	1.51E-09
Bt.29544.1.A1_at	*ODF2L*	outer dense fiber of sperm tails 2-like	4.392	6.14E-06

Bt.17777.2.S1_at	*OPTN*	optineurin	6.494	4.80E-06
Bt.17777.3.S1_at	*OPTN*	optineurin	6.331	2.94E-06
Bt.17777.1.S1_at	*OPTN*	optineurin	3.834	4.89E-05
Bt.27953.1.S1_at	*OSBPL8*	oxysterol binding protein-like 8	3.084	4.00E-08
Bt.1087.1.S1_at	*PAPSS2*	3'-phosphoadenosine 5'-phosphosulfate synthase 2	-7.761	1.30E-07
Bt.19503.1.A1_at	*PARD3B*	par-3 partitioning defective 3 homolog B (C. elegans)	3.198	7.35E-06
Bt.27072.1.A1_at	*PBRM1*	polybromo 1	4.259	2.65E-07
Bt.7955.2.S1_at	*PCOLCE*	procollagen C-endopeptidase enhancer	5.738	5.58E-09
Bt.7955.1.S1_at	*PCOLCE*	procollagen C-endopeptidase enhancer	3.953	8.06E-10
Bt.4770.1.S1_at	*PDCD4*	programmed cell death 4 (neoplastic transformation inhibitor)	3.977	4.19E-04
Bt.3014.1.A1_at	*PDGFC*	platelet derived growth factor C	-4.55	3.71E-08
Bt.1358.1.S1_at	*PDLIM1*	PDZ and LIM domain 1	-3.291	1.53E-03
Bt.10813.1.S1_at	*PDLIM4*	PDZ and LIM domain 4	5.004	4.00E-05

Bt.11058.1.A1_at	*PDP1*	pyruvate dehyrogenase phosphatase catalytic subunit 1	3.233	7.83E-08
Bt.9459.1.S1_at	*PDPK1*	3-phosphoinositide dependent protein kinase-1	4.532	1.89E-05
Bt.22306.1.S1_at	*PDSS1*	prenyl (decaprenyl) diphosphate synthase, subunit 1	5.995	1.59E-06
Bt.12314.1.S1_at	*PFKFB3*	6-phosphofructo-2-kinase/fructose-2,6-biphosphatase 3	-3.165	5.81E-04
Bt.2347.1.S1_at	*PFKM*	phosphofructokinase, muscle	3.674	1.81E-08
Bt.13036.1.S1_at	*PGR*	progesterone receptor	4.676	9.13E-08
Bt.15306.1.A1_at	*PHF3*	PHD finger protein 3	3.068	5.00E-04
Bt.4733.1.S1_at	*PHGDH*	phosphoglycerate dehydrogenase	-3.681	2.23E-04
Bt.12572.2.S1_at	*PIGS*	phosphatidylinositol glycan anchor biosynthesis, class S	5.454	3.21E-06
Bt.7236.1.S1_at	*PIK3R1*	phosphoinositide-3-kinase, regulatory subunit 1 (alpha)	8.953	4.64E-07
Bt.9193.1.S1_at	*PIK3R1*	phosphoinositide-3-kinase, regulatory subunit 1 (alpha)	4.246	5.77E-07
Bt.18751.2.A1_s_at	*PIP4K2A*	phosphatidylinositol-5-phosphate 4-kinase, type II, alpha	3.111	6.17E-04
Bt.12302.1.S1_at	*PLAT*	plasminogen activator, tissue	17.462	8.50E-07

Bt.13996.3.A1_at	*PLD1*	phospholipase D1, phosphatidylcholine-specific	3.731	1.17E-07
Bt.22283.1.S1_at	*PLEKHA2*	pleckstrin homology domain containing, family A (phosphoinositide binding specific) member 2	4.174	5.43E-06
Bt.8690.1.S1_at	*PLEKHB2*	pleckstrin homology domain containing, family B (evectins) member 2	3.47	6.46E-05
Bt.18945.1.A1_at	*PLEKHG1*	pleckstrin homology domain containing, family G (with RhoGef domain) member 1	3.625	1.73E-08
Bt.12361.1.S1_at	*PLEKHH3*	pleckstrin homology domain containing, family H (with MyTH4 domain) member 3	4.143	8.65E-09
Bt.3323.2.S1_at	*PLXNB2*	plexin B2	4.72	1.27E-04
Bt.26968.1.S1_at	*PLXNC1*	plexin C1	4.826	1.10E-04
Bt.6608.1.S1_at	*PLXNC1*	plexin C1	3.4	6.49E-05
Bt.20878.1.A1_at	*PNISR*	PNN-interacting serine/arginine-rich protein	3.666	2.19E-03
Bt.4696.1.S1_at	*POR*	P450 (cytochrome) oxidoreductase	3.321	1.02E-05
Bt.23509.1.S1_at	*PPAP2B*	phosphatidic acid phosphatase type 2B	3.725	2.37E-05
Bt.18634.2.S1_at	*PPM1K*	protein phosphatase, Mg2+/Mn2+ dependent, 1K	5.94	8.85E-07
Bt.18634.1.A1_at	*PPM1K*	protein phosphatase, Mg2+/Mn2+ dependent, 1K	4.558	3.09E-07

Bt.21997.2.A1_at	*PPM1K*	protein phosphatase, Mg2+/Mn2+ dependent, 1K	4.495	3.52E-06
Bt.21997.1.S1_at	*PPM1K*	protein phosphatase, Mg2+/Mn2+ dependent, 1K	3.762	1.33E-05
Bt.1549.2.S1_at	*PPP2R5E*	protein phosphatase 2, regulatory subunit B', epsilon isoform	3.008	1.13E-04
Bt.15677.1.A1_at	*PRPF38B*	PRP38 pre-mRNA processing factor 38 (yeast) domain containing B	4.229	1.74E-04
Bt.20330.1.S1_at	*PRSS23*	protease, serine, 23	48.455	2.73E-10
Bt.24364.1.A1_at	*PRSS35*	protease, serine, 35	-3.694	6.29E-05
Bt.5467.1.S1_at	*PSAP*	prosaposin	3.732	3.48E-04
Bt.12570.1.S1_at	*PSMD4*	proteasome (prosome, macropain) 26S subunit, non-ATPase, 4	3.799	8.56E-05
Bt.352.2.S1_at	*PTGFR*	prostaglandin F receptor (FP)	5.48	4.41E-05
Bt.15758.1.S1_at	*PTGS2*	prostaglandin-endoperoxide synthase 2 (prostaglandin G/H synthase and cyclooxygenase)	-3.547	3.76E-03
Bt.12848.1.S1_at	*PTHLH*	parathyroid hormone-like hormone	12.825	3.32E-12
Bt.7024.1.A1_at	*PTP4A2*	protein tyrosine phosphatase type IVA, member 2	3.084	2.95E-05
Bt.19859.1.S1_at	*PTPN11*	protein tyrosine phosphatase, non-receptor type 11	3.072	1.18E-04

Bt.5098.1.S1_at	*PTPN13*	protein tyrosine phosphatase, non-receptor type 13 (APO-1/CD95 (Fas)-associated phosphatase)	4.211	4.17E-09
Bt.7538.1.S1_at	*PTPRN2*	protein tyrosine phosphatase, receptor type, N polypeptide 2	-3.572	1.09E-03
Bt.23682.1.S1_at	*PYGL*	phosphorylase, glycogen, liver	8.851	2.22E-05
Bt.28093.1.S1_at	*PYGL*	phosphorylase, glycogen, liver	5.699	7.66E-05
Bt.17714.1.A1_at	*PYGL*	phosphorylase, glycogen, liver	3.62	3.31E-03
Bt.23098.1.S1_at	*QSOX1*	quiescin Q6 sulfhydryl oxidase 1	3.521	1.47E-05
Bt.4379.1.S1_at	*R3HCC1*	R3H domain and coiled-coil containing 1	4.247	8.64E-06
Bt.2741.1.A1_at	*RAB7A*	RAB7A, member RAS oncogene family	3.269	3.47E-06
Bt.27405.2.S1_at	*RAD50*	RAD50 homolog (S. cerevisiae)	3.115	8.17E-04
Bt.21170.1.A1_at	*RASA2*	RAS p21 protein activator 2	5.516	2.49E-07
Bt.28723.1.A1_at	*RASL11B*	RAS-like, family 11, member B	-6.121	1.55E-04
Bt.1974.1.S1_at	*RASL11B*	RAS-like, family 11, member B	-6.784	3.30E-04
Bt.3013.1.S1_a_at	*RBFOX2*	RNA binding protein, fox-1 homolog (C. elegans) 2	3.434	2.83E-05

Bt.17614.2.A1_at	*RBM25*	RNA binding motif protein 25	4.434	4.02E-06
Bt.27798.2.S1_at	*RBM5*	RNA binding motif protein 5	3.992	2.94E-05
Bt.27798.1.A1_at	*RBM5*	RNA binding motif protein 5	3.175	7.90E-03
Bt.16273.1.A1_at	*RCAN3*	RCAN family member 3	4.08	1.10E-06
Bt.4078.2.S1_a_at	*RCN3*	reticulocalbin 3, EF-hand calcium binding domain	3.603	8.77E-04
Bt.18951.2.S1_at	*RDH11*	retinol dehydrogenase 11 (all-trans/9-cis/11-cis)	3.126	2.93E-04
Bt.2629.1.S1_at	*RENBP*	renin binding protein	-3.564	4.57E-07
Bt.502.1.A1_at	*RGN*	regucalcin (senescence marker protein-30)	9.804	1.57E-10
Bt.4245.3.S1_a_at	*RHBDD2*	rhomboid domain containing 2	3.778	4.39E-06
Bt.4245.2.S1_a_at	*RHBDD2*	rhomboid domain containing 2	3.45	6.95E-07
Bt.4245.1.S1_at	*RHBDD2*	rhomboid domain containing 2	3.367	2.23E-06
Bt.4634.2.S1_a_at	*RNASEK*	ribonuclease, RNase K	3.588	4.27E-06
Bt.27401.1.A1_at	*RNF128*	ring finger protein 128, E3 ubiquitin protein ligase	4.487	5.29E-05

Bt.23857.1.A1_at	*RNF144B*	ring finger protein 144B	4.253	1.73E-06
Bt.10397.2.S1_at	*RNF20*	ring finger protein 20, E3 ubiquitin protein ligase	3.273	3.86E-05
Bt.9179.2.S1_at	*RNF213*	ring finger protein 213	5.901	2.56E-09
Bt.9179.1.A1_at	*RNF213*	ring finger protein 213	5.398	4.97E-08
Bt.23672.1.A1_at	*ROBO1*	roundabout, axon guidance receptor, homolog 1 (Drosophila)	3.125	1.62E-02
Bt.4177.3.A1_at	*ROBO2*	roundabout, axon guidance receptor, homolog 2 (Drosophila)	6.684	9.97E-09
Bt.16332.1.A1_at	*ROBO2*	roundabout, axon guidance receptor, homolog 2 (Drosophila)	4.602	7.67E-09
Bt.120.2.S1_at	*ROCK1*	Rho-associated, coiled-coil containing protein kinase 1	3.117	1.02E-03
Bt.22279.1.S1_at	*RPRM*	reprimo, TP53 dependent G2 arrest mediator candidate	-5.795	4.48E-04
Bt.24973.1.A1_at	*Rrbp1*	ribosome binding protein 1	4.156	1.31E-04
Bt.22064.2.S1_at	*RSRC2*	arginine/serine-rich coiled-coil 2	5.136	8.11E-05
Bt.11418.1.S1_at	*RYK*	receptor-like tyrosine kinase	4.179	2.73E-08
Bt.8374.1.S1_at	*RYR2*	ryanodine receptor 2 (cardiac)	-3.021	6.34E-05

Bt.27212.1.A1_at	*RYR2*	ryanodine receptor 2 (cardiac)	-6.965	1.73E-04
Bt.16994.2.A1_at	*SAFB2*	scaffold attachment factor B2	4.887	4.22E-04
Bt.20240.1.S1_at	*SASH1*	SAM and SH3 domain containing 1	-3.458	3.19E-04
Bt.13249.1.A1_at	*SCD5*	stearoyl-CoA desaturase 5	4.175	1.55E-06
Bt.5427.1.A1_at	*SCG2*	secretogranin II	3.156	1.95E-02
Bt.27476.1.S1_at	*SCYL2*	SCY1-like 2 (S. cerevisiae)	4.041	9.10E-06
Bt.2494.2.S1_a_at	*SDC2*	syndecan 2	3.782	1.42E-04
Bt.13419.2.A1_at	*SEC63*	SEC63 homolog (S. cerevisiae)	3.433	6.94E-06
Bt.24923.1.S1_at	*SEL1L3*	sel-1 suppressor of lin-12-like 3 (C. elegans)	-3.554	5.36E-07
Bt.17078.1.S1_at	*SEMA6A*	sema domain, transmembrane domain (TM), and cytoplasmic domain, (semaphorin) 6A	3.283	1.12E-08
Bt.19862.2.S1_at	*SEMA6D*	sema domain, transmembrane domain (TM), and cytoplasmic domain, (semaphorin) 6D	6.157	1.56E-05
Bt.19862.1.A1_at	*SEMA6D*	sema domain, transmembrane domain (TM), and cytoplasmic domain, (semaphorin) 6D	4.367	1.95E-06
Bt.14150.1.S1_at	*SEPT4*	septin 4	-15.311	1.87E-06

Bt.2712.1.S1_at	*SERPINA5*	serpin peptidase inhibitor, clade A (alpha-1 antiproteinase, antitrypsin), member 5	11.154	7.61E-07
Bt.27429.1.S1_at	*SF3B1*	splicing factor 3b, subunit 1, 155kDa	4.583	6.03E-06
Bt.27429.2.A1_at	*SF3B1*	splicing factor 3b, subunit 1, 155kDa	3.083	4.23E-06
Bt.3540.1.S1_at	*SFRP4*	secreted frizzled-related protein 4	-3.012	2.80E-03
Bt.12396.1.S1_at	*SGSH*	N-sulfoglucosamine sulfohydrolase	3.633	2.23E-09
Bt.2215.1.S1_at	*SH3GL2*	SH3-domain GRB2-like 2	4.127	1.93E-07
Bt.22389.1.S1_at	*SHISA2*	shisa homolog 2 (Xenopus laevis)	-3.471	4.60E-03
Bt.2989.3.S1_at	*SLC25A12*	solute carrier family 25 (aspartate/glutamate carrier), member 12	3.582	1.10E-05
Bt.20517.1.S1_at	*SLC25A28*	solute carrier family 25 (mitochondrial iron transporter), member 28	5.817	1.82E-08
Bt.8620.1.S1_at	*SLC26A11*	solute carrier family 26, member 11	3.577	6.19E-09
Bt.1828.1.A1_at	*SLC27A3*	solute carrier family 27 (fatty acid transporter), member 3	5.692	1.45E-04
Bt.22697.1.A1_at	*SLC39A8*	solute carrier family 39 (zinc transporter), member 8	9.059	1.36E-05
Bt.22759.1.S1_at	*SLC40A1*	solute carrier family 40 (iron-regulated transporter), member 1	5.339	9.25E-04

Bt.3187.1.A1_s_at	*SLC5A11*	solute carrier family 5 (sodium/glucose cotransporter), member 11	15.634	2.74E-11
Bt.25438.1.A1_at	*SLC5A11*	solute carrier family 5 (sodium/glucose cotransporter), member 11	3.995	4.69E-11
Bt.17460.1.A1_at	*SLITRK2*	SLIT and NTRK-like family, member 2	5.126	2.46E-04
Bt.14123.1.S1_at	*SLMAP*	sarcolemma associated protein	5.114	9.19E-10
Bt.13371.1.S1_at	*SNTB2*	syntrophin, beta 2 (dystrophin-associated protein A1, 59kDa, basic component 2)	3.392	2.51E-06
Bt.26309.1.A1_at	*SNX31*	sorting nexin 31	-5.976	1.86E-07
Bt.11239.3.S1_at	*SPG7*	spastic paraplegia 7 (pure and complicated autosomal recessive)	4.227	3.39E-06
Bt.2520.1.S1_at	*SPOCK2*	sparc/osteonectin, cwcv and kazal-like domains proteoglycan (testican) 2	21.995	8.31E-08
Bt.27816.1.S1_at	*STAC3*	SH3 and cysteine rich domain 3	-3.911	1.41E-05
Bt.4709.1.S1_at	*STAR*	steroidogenic acute regulatory protein	3.389	4.06E-02
Bt.1920.1.S1_at	*STARD10*	StAR-related lipid transfer (START) domain containing 10	-3.328	6.24E-05
Bt.1920.2.S1_at	*STARD10*	StAR-related lipid transfer (START) domain containing 10	-3.397	2.01E-05
Bt.8441.2.S1_at	*STBD1*	starch binding domain 1	3.09	2.54E-03

Bt.8712.1.S1_at	*STIM1*	stromal interaction molecule 1	3.195	2.16E-05
Bt.3196.2.S1_a_at	*STRA6*	stimulated by retinoic acid gene 6 homolog (mouse)	4.215	3.81E-03
Bt.29837.1.S1_at	*SUSD4*	sushi domain containing 4	3.539	2.37E-03
Bt.29086.1.S1_at	*SVOPL*	SVOP-like	-8.557	1.68E-03
Bt.9438.1.S1_at	*SYNCRIP*	synaptotagmin binding, cytoplasmic RNA interacting protein	3.649	4.63E-04
Bt.8220.2.S1_at	*TACC1*	transforming, acidic coiled-coil containing protein 1	3.507	1.52E-05
Bt.8220.3.S1_at	*TACC1*	transforming, acidic coiled-coil containing protein 1	3.146	1.92E-06
Bt.16732.1.A1_at	*TBC1D5*	TBC1 domain family, member 5	3.043	2.25E-04
Bt.15811.1.A1_at	*TBCEL*	tubulin folding cofactor E-like	4.813	3.27E-08
Bt.15811.3.S1_at	*TBCEL*	tubulin folding cofactor E-like	3.383	2.14E-07
Bt.6824.1.A1_at	*TET2*	tet methylcytosine dioxygenase 2	3.695	2.62E-05
Bt.17646.2.S1_at	*TFCP2*	transcription factor CP2	3.056	9.69E-07
Bt.6186.1.S1_at	*TFR2*	transferrin receptor 2	3.312	1.21E-05

Bt.20778.2.S1_at	*TGIF1*	TGFB-induced factor homeobox 1	-3.171	2.51E-06
Bt.20778.1.S1_at	*TGIF1*	TGFB-induced factor homeobox 1	-3.497	2.17E-08
Bt.16329.1.A1_at	*TGIF1*	TGFB-induced factor homeobox 1	-6.141	1.26E-07
Bt.3011.1.A1_at	*THBS3*	thrombospondin 3	3.532	2.83E-09
Bt.3011.2.S1_at	*THBS3*	thrombospondin 3	3.282	1.70E-06
Bt.29472.1.A1_at	*TIA1*	TIA1 cytotoxic granule-associated RNA binding protein	3.918	1.61E-05
Bt.632.1.S1_s_at	*TIMP1*	TIMP metallopeptidase inhibitor 1	4.067	4.31E-03
Bt.14176.2.S1_at	*TIMP2*	TIMP metallopeptidase inhibitor 2	7.9	1.13E-05
Bt.435.1.S1_at	*TIMP2*	TIMP metallopeptidase inhibitor 2	6.703	6.24E-06
Bt.14176.1.S1_at	*TIMP2*	TIMP metallopeptidase inhibitor 2	5.444	3.58E-06
Bt.26582.1.S1_at	*TM2D1*	TM2 domain containing 1	3.071	1.16E-09
Bt.28082.1.S1_at	*TM9SF4*	transmembrane 9 superfamily protein member 4	3.536	5.14E-09
Bt.16392.2.A1_at	*TM9SF4*	transmembrane 9 superfamily protein member 4	3.178	1.49E-05

Bt.16392.1.S1_at	*TM9SF4*	transmembrane 9 superfamily protein member 4	3.12	9.32E-05
Bt.15565.1.S1_at	*TMEM176A*	transmembrane protein 176A	4.071	7.64E-05
Bt.21250.1.S1_a_at	*TMEM176B*	transmembrane protein 176B	3.677	2.17E-06
Bt.21824.1.A1_at	*TMEM27*	transmembrane protein 27	5.141	9.99E-08
Bt.26553.1.A1_at	*TMEM47*	transmembrane protein 47	4.549	6.05E-08
Bt.13762.1.S1_at	*TMEM50B*	transmembrane protein 50B	3.238	2.44E-06
Bt.7923.1.A1_at	*TMIGD2*	transmembrane and immunoglobulin domain containing 2	-3.006	1.53E-05
Bt.9570.1.S1_at	*TNFAIP2*	tumor necrosis factor, alpha-induced protein 2	-17.49	1.32E-04
Bt.958.1.A1_at	*TNFAIP6*	tumor necrosis factor, alpha-induced protein 6	279.55	4.36E-09
Bt.958.2.S1_at	*TNFAIP6*	tumor necrosis factor, alpha-induced protein 6	83.153	4.62E-10
Bt.26969.1.A1_at	*TNFAIP8L3*	tumor necrosis factor, alpha-induced protein 8-like 3	4.654	2.76E-07
Bt.14438.2.A1_a_at	*TNPO1*	transportin 1	5.544	1.22E-04
Bt.22975.1.S1_at	*TNPO1*	transportin 1	3.512	1.93E-04

Bt.2399.1.S1_at	*TNS3*	tensin 3	3.629	2.22E-08
Bt.27061.1.S1_at	*TOB1*	transducer of ERBB2, 1	6.264	4.12E-08
Bt.15703.2.S1_at	*TOPORS*	topoisomerase I binding, arginine/serine-rich, E3 ubiquitin protein ligase	3.321	7.36E-05
Bt.8400.1.A1_at	*TOX*	thymocyte selection-associated high mobility group box	11.433	5.23E-09
Bt.16524.1.A1_at	*TPR*	translocated promoter region, nuclear basket protein	4.298	5.97E-08
Bt.22857.1.S1_at	*TRIB2*	tribbles homolog 2 (Drosophila)	3.202	2.05E-02
Bt.13750.1.A1_at	*TRIM2*	tripartite motif containing 2	-3.334	2.41E-06
Bt.13869.1.A1_at	*TRIM25*	tripartite motif containing 25	3.725	4.60E-07
Bt.24341.1.S1_at	*TSPAN2*	tetraspanin 2	3.022	2.97E-08
Bt.24478.1.S1_at	*TSPAN9*	tetraspanin 9	3.436	5.32E-10
Bt.10920.1.S1_at	*TTC3*	tetratricopeptide repeat domain 3	3.136	4.47E-04
Bt.754.1.S1_at	*TTLL3*	tubulin tyrosine ligase-like family, member 3	3.886	4.13E-05
Bt.21767.1.S1_a_at	*TTN*	titin	-3.573	7.19E-05

Bt.12327.1.S1_at	*TXNIP*	thioredoxin interacting protein	4.136	1.88E-05
Bt.8857.1.S1_at	*UBQLN1*	ubiquilin 1	3.009	8.37E-04
Bt.26936.1.S1_at	*UBR1*	ubiquitin protein ligase E3 component n-recognin 1	3.962	2.42E-05
Bt.28146.1.A1_at	*UBR3*	ubiquitin protein ligase E3 component n-recognin 3 (putative)	3.193	2.61E-06
Bt.25887.1.A1_at	*UGCG*	UDP-glucose ceramide glucosyltransferase	3.984	1.66E-06
Bt.29411.1.S1_at	*UHRF1BP1L*	UHRF1 binding protein 1-like	3.482	3.66E-06
Bt.22118.1.A1_at	*USHBP1*	Usher syndrome 1C binding protein 1	5.225	1.08E-08
Bt.22118.3.S1_at	*USHBP1*	Usher syndrome 1C binding protein 1	3.553	2.20E-10
Bt.17774.1.A1_at	*USP4*	ubiquitin specific peptidase 4 (proto-oncogene)	3.982	1.48E-07
Bt.21986.1.A1_a_at	*USP7*	ubiquitin specific peptidase 7 (herpes virus-associated)	6.414	2.15E-05
Bt.21986.1.A1_at	*USP7*	ubiquitin specific peptidase 7 (herpes virus-associated)	4.019	6.77E-06
Bt.20427.2.S1_at	*UTP6*	UTP6, small subunit (SSU) processome component, homolog (yeast)	4.807	9.99E-05
Bt.7043.2.S1_a_at	*VCAM1*	vascular cell adhesion molecule 1	7.293	3.17E-05

Bt.5395.1.S1_a_at	*VCAN*	versican	4.99	3.17E-04
Bt.6057.1.S1_at	*VWA1*	von Willebrand factor A domain containing 1	3.021	1.84E-04
Bt.26722.1.A1_a_at	*WDFY4*	WDFY family member 4	3.88	7.93E-06
Bt.24284.1.A1_at	*WHSC1L1*	Wolf-Hirschhorn syndrome candidate 1-like 1	3.912	6.02E-04
Bt.27247.1.A1_at	*WHSC1L1*	Wolf-Hirschhorn syndrome candidate 1-like 1	3.046	1.32E-03
Bt.26825.2.S1_at	*XRN2*	5'-3' exoribonuclease 2	3.129	1.04E-04
Bt.5748.3.S1_at	*YPEL5*	yippee-like 5 (Drosophila)	3.457	1.31E-05
Bt.5748.2.S1_at	*YPEL5*	yippee-like 5 (Drosophila)	3.138	1.61E-05
Bt.25188.1.A1_at	*ZBTB33*	zinc finger and BTB domain containing 33	3.013	7.30E-07
Bt.1075.1.A1_at	*ZNF24*	zinc finger protein 24	3.259	1.76E-05
Bt.27394.1.A1_at	*ZNF292*	zinc finger protein 292	6.011	5.13E-05
Bt.17868.1.A1_at	*ZNF317*	zinc finger protein 317	3.819	1.01E-05
Bt.26259.3.A1_a_at	*ZNF462*	zinc finger protein 462	3.134	6.66E-05

Bt.9496.1.S1_at	*ZNF609*	zinc finger protein 609	3.573	7.04E-06
Bt.511.1.S1_at	*ZP3*	zona pellucida glycoprotein 3 (sperm receptor)	-3.151	2.90E-05
Bt.22477.1.S1_at			33.853	3.28E-09
Bt.23113.1.A1_at			15.489	3.55E-11
Bt.1929.1.S1_at			15.127	3.29E-08
Bt.28648.1.S1_at			12.366	2.82E-06
Bt.25248.1.A1_at			11.841	1.42E-09
Bt.21929.1.S1_at			10.361	3.43E-07
Bt.17821.3.A1_at			10.244	1.07E-05
Bt.16628.2.A1_at			10.139	6.06E-04
Bt.24348.1.A1_at			9.804	4.24E-07
Bt.17821.1.S1_at			8.317	2.49E-07
Bt.24701.1.A1_at			7.712	2.31E-07

Bt.15298.1.A1_at			7.646	5.76E-05
Bt.19217.1.A1_at			7.639	2.55E-07
Bt.5713.1.A1_at			7.637	2.25E-10
Bt.24940.1.A1_at			7.465	2.01E-03
Bt.16628.1.S1_at			7.366	4.29E-05
Bt.27285.1.S1_at			7.229	1.08E-03
Bt.26299.1.A1_at			7.18	1.03E-03
Bt.21373.2.A1_at			7.031	2.76E-11
Bt.4943.3.S1_a_at			6.912	1.02E-06
Bt.10361.1.S1_at			6.796	7.66E-09
Bt.20659.1.S1_at			6.729	2.07E-10
Bt.6892.1.S1_at			6.6	9.69E-07
Bt.22121.1.A1_at			6.557	2.06E-06

Bt.27145.1.A1_at			6.491	3.78E-04
Bt.15278.1.A1_at			6.453	4.86E-08
Bt.24958.1.S1_at			6.327	3.15E-08
Bt.15442.1.A1_at			6.309	2.61E-05
Bt.12294.1.S1_at			5.997	5.54E-06
Bt.6312.1.S1_at			5.861	6.73E-08
Bt.25196.1.A1_at			5.768	1.64E-05
Bt.25637.1.A1_at			5.635	1.34E-07
Bt.11150.1.S1_at			5.479	2.63E-04
Bt.22627.1.A1_at			5.465	4.44E-06
Bt.18857.1.A1_at			5.453	1.33E-11
Bt.24797.1.A1_at			5.451	2.41E-06
Bt.26242.1.A1_at			5.429	6.99E-05

Bt.28292.2.S1_a_at			5.424	5.93E-07
Bt.17387.1.A1_at			5.408	1.34E-06
Bt.1451.1.S1_at			5.329	1.25E-06
Bt.24997.1.S1_at			5.29	1.31E-07
Bt.10994.1.S1_at			5.153	4.71E-05
Bt.21956.2.A1_at			5.124	2.82E-09
Bt.24319.1.A1_at			5.119	1.29E-10
Bt.18671.1.A1_at			4.85	5.55E-09
Bt.5856.1.S1_at			4.809	1.02E-09
Bt.23555.1.S1_at			4.807	3.17E-07
AFFX-ThrX-5_at			4.796	1.29E-03
Bt.1705.1.S1_at			4.652	1.43E-08
Bt.2099.1.S1_at			4.64	7.97E-07

Bt.17713.2.A1_s_at			4.637	1.44E-05
Bt.12199.2.S1_at			4.598	8.01E-07
Bt.18184.1.A1_at			4.574	2.86E-08
Bt.9925.1.S1_at			4.498	7.87E-09
Bt.20658.1.A1_at			4.456	4.61E-08
Bt.12574.1.A1_at			4.441	4.68E-06
Bt.24262.1.S1_at			4.415	2.31E-04
Bt.15788.1.S1_a_at			4.406	1.49E-03
Bt.23205.1.S1_s_at			4.398	1.90E-05
Bt.1742.2.S1_a_at			4.328	7.32E-04
Bt.23555.2.S1_at			4.313	8.21E-07
Bt.20605.1.S1_at			4.307	8.78E-03
Bt.18520.1.A1_at			4.289	7.93E-06

Bt.2357.1.S1_at			4.287	1.53E-04
Bt.24391.1.A1_at			4.241	2.67E-04
Bt.7839.1.A1_at			4.235	1.25E-04
Bt.23297.1.A1_at			4.211	1.98E-06
Bt.3253.3.S1_at			4.204	7.28E-06
Bt.16573.1.A1_at			4.176	2.81E-04
Bt.3253.1.A1_at			4.144	8.57E-06
Bt.11599.1.S1_at			4.137	1.68E-08
Bt.17988.2.A1_at			4.103	2.03E-04
Bt.17373.2.A1_at			4.068	2.99E-05
Bt.25915.1.A1_at			4.038	4.36E-10
Bt.22756.1.A1_at			4.013	4.08E-05
AFFX-PheX-5_at			4.005	5.27E-04

Bt.18551.1.S1_at			3.977	3.86E-06
Bt.18167.1.A1_at			3.958	8.99E-05
Bt.2465.1.S1_at			3.947	2.18E-05
Bt.10044.1.A1_at			3.927	9.86E-06
Bt.13203.1.S1_at			3.882	4.45E-03
Bt.6784.1.A1_at			3.838	1.56E-03
Bt.5713.2.S1_at			3.826	4.09E-09
Bt.22117.1.S1_at			3.805	3.31E-04
Bt.17026.2.A1_at			3.785	4.79E-04
Bt.22594.2.S1_a_at			3.769	1.68E-06
Bt.24260.2.S1_at			3.759	1.52E-07
Bt.22467.1.S1_at			3.757	1.81E-06
Bt.20598.1.S1_at			3.747	8.50E-10

Bt.3589.2.A1_at			3.746	2.58E-07
Bt.17760.1.A1_at			3.727	2.41E-05
Bt.16793.1.A1_at			3.717	6.49E-06
Bt.6848.1.S1_at			3.717	3.22E-03
Bt.28530.1.A1_at			3.692	1.45E-05
Bt.25338.1.S1_at			3.649	1.43E-09
Bt.24105.1.A1_at			3.634	3.08E-06
Bt.5600.1.A1_at			3.632	2.68E-04
Bt.13495.1.S1_at			3.629	3.38E-04
Bt.28238.1.A1_at			3.624	3.68E-06
Bt.20899.1.A1_at			3.617	2.79E-03
Bt.13993.2.S1_at			3.598	2.79E-08
Bt.19382.1.S1_at			3.579	1.10E-05

Bt.6863.1.A1_at			3.579	2.37E-04
Bt.27206.1.A1_at			3.557	4.51E-05
Bt.24457.1.S1_at			3.532	7.46E-05
Bt.1174.1.S1_at			3.506	3.51E-07
Bt.25240.1.A1_at			3.501	9.97E-04
Bt.21956.1.S1_at			3.5	7.03E-09
Bt.15231.1.S1_at			3.488	3.11E-07
Bt.18271.1.A1_at			3.465	8.62E-05
Bt.19004.2.A1_at			3.454	1.79E-05
Bt.16241.1.A1_at			3.453	6.61E-06
Bt.26086.1.S1_at			3.447	5.77E-09
Bt.11778.1.A1_at			3.445	2.49E-03
Bt.4870.1.S1_at			3.422	2.21E-05

Bt.25226.1.A1_at			3.418	5.56E-08
Bt.12203.1.S1_at			3.385	3.03E-05
Bt.23673.1.A1_at			3.381	2.66E-03
Bt.15540.1.A1_at			3.356	5.76E-07
Bt.13229.1.S1_at			3.353	7.48E-05
Bt.10101.1.S1_at			3.353	2.51E-07
Bt.12360.1.S1_at			3.315	1.08E-07
Bt.6017.1.A1_at			3.291	3.44E-05
Bt.17129.2.A1_at			3.282	1.00E-07
Bt.14204.1.A1_at			3.28	3.85E-05
Bt.24328.1.S1_at			3.266	2.43E-06
Bt.16234.2.S1_at			3.25	3.67E-04
Bt.13538.2.S1_at			3.24	8.69E-07

Bt.24260.1.S1_at			3.236	4.61E-07
Bt.22018.2.S1_at			3.224	1.23E-05
Bt.22033.1.A1_at			3.223	1.19E-04
Bt.5869.1.S1_at			3.208	2.95E-04
Bt.22594.1.S1_at			3.204	8.17E-05
Bt.7816.1.S1_at			3.192	6.22E-08
Bt.26869.1.A1_at			3.183	4.99E-04
Bt.17577.1.A1_at			3.171	3.35E-04
Bt.23980.1.A1_at			3.134	4.88E-03
Bt.19004.1.S1_at			3.127	1.14E-05
Bt.26117.1.A1_at			3.111	2.23E-05
Bt.23418.1.S1_at			3.104	1.68E-02
Bt.8317.1.S1_at			3.093	4.91E-04

Bt.12032.1.S1_at			3.089	1.54E-07
Bt.10222.1.S1_at			3.083	3.27E-06
Bt.18794.1.S1_at			3.07	1.96E-11
Bt.19496.1.A1_at			3.059	2.22E-03
Bt.18130.1.A1_at			3.053	1.67E-05
Bt.22049.1.S1_at			3.052	2.18E-06
Bt.13538.1.A1_at			3.049	2.79E-06
Bt.19203.1.A1_at			3.045	5.37E-04
Bt.23747.1.A1_at			3.037	4.16E-09
Bt.27797.1.A1_at			3.033	4.70E-04
Bt.25676.1.A1_at			3.029	1.08E-05
Bt.17821.2.S1_at			3.026	2.40E-04
Bt.944.1.S1_at			3.025	1.52E-05

Bt.10893.1.S1_at			3.017	1.64E-03
Bt.9538.1.S1_at			3.001	1.05E-03
Bt.26011.1.A1_at			-3.006	8.89E-05
Bt.27543.1.A1_at			-3.01	1.83E-07
Bt.8875.1.A1_at			-3.022	2.30E-05
Bt.29194.1.S1_at			-3.038	1.70E-08
Bt.13003.15.A1_at			-3.04	6.39E-06
Bt.19567.2.S1_at			-3.046	3.62E-04
Bt.21190.1.A1_at			-3.051	2.78E-05
Bt.13735.1.S1_at			-3.065	2.37E-06
Bt.9601.1.S1_at			-3.069	2.20E-04
Bt.25356.1.A1_at			-3.08	7.09E-06
Bt.11696.1.A1_at			-3.117	2.16E-03

Bt.15568.1.A1_at			-3.117	1.12E-04
Bt.8618.1.S1_at			-3.123	1.04E-05
Bt.19567.1.A1_at			-3.132	1.75E-04
Bt.29593.1.A1_at			-3.135	1.07E-05
Bt.18632.1.A1_at			-3.138	1.47E-04
Bt.19123.1.A1_at			-3.158	7.75E-06
Bt.23492.1.S1_at			-3.17	5.26E-04
Bt.21847.1.A1_at			-3.183	2.21E-05
Bt.25110.1.A1_at			-3.218	1.22E-04
Bt.28382.1.S1_at			-3.225	1.10E-06
Bt.14014.1.S1_at			-3.231	6.71E-04
Bt.28515.1.A1_at			-3.263	1.02E-04
Bt.29530.1.S1_at			-3.266	4.27E-05

Bt.10922.1.A1_at			-3.266	2.29E-04
Bt.28767.1.A1_at			-3.308	5.09E-05
Bt.24179.1.A1_at			-3.312	5.23E-04
Bt.20994.1.S1_at			-3.323	2.95E-06
Bt.29845.1.S1_at			-3.343	5.90E-05
Bt.2537.2.S1_at			-3.355	1.22E-05
Bt.8909.1.S1_at			-3.356	1.00E-05
Bt.20093.1.S1_at			-3.358	9.48E-04
Bt.10785.1.A1_at			-3.367	9.36E-05
Bt.10855.1.S1_at			-3.372	1.54E-02
Bt.24868.1.A1_at			-3.408	2.29E-04
Bt.28307.1.S1_at			-3.425	1.43E-04
Bt.23889.1.A1_at			-3.588	9.66E-05

Bt.17085.1.A1_at			-3.605	5.13E-05
Bt.4981.1.S1_at			-3.666	2.02E-03
Bt.29494.1.A1_at			-3.668	1.13E-05
Bt.20744.1.A1_at			-3.684	8.81E-09
Bt.16333.1.A1_at			-3.701	2.26E-05
Bt.16109.1.S1_at			-3.707	5.18E-07
Bt.3213.1.A1_at			-3.709	3.99E-05
Bt.27687.1.A1_at			-3.733	2.86E-05
Bt.22200.1.S1_at			-3.752	2.92E-06
Bt.19354.1.A1_at			-3.766	2.46E-05
Bt.6376.1.A1_at			-3.795	1.07E-08
Bt.29752.1.A1_at			-3.803	1.01E-04
Bt.22670.2.S1_at			-3.993	9.91E-07

Bt.3590.1.A1_at			-3.998	2.08E-04
Bt.17853.1.A1_at			-4.009	2.86E-05
Bt.7389.2.A1_at			-4.054	4.41E-05
Bt.4389.1.S1_at			-4.071	2.93E-06
Bt.28960.1.A1_at			-4.19	2.45E-02
Bt.18311.1.A1_at			-4.322	3.45E-06
Bt.27654.1.S1_at			-4.382	3.17E-11
Bt.13003.10.A1_at			-4.453	3.61E-06
Bt.5154.1.S1_at			-4.46	2.05E-06
Bt.3782.1.A1_at			-4.602	9.87E-04
Bt.18774.1.A1_at			-4.603	1.77E-05
Bt.28332.1.S1_at			-4.895	5.33E-08
Bt.27457.1.S1_at			-5.338	2.27E-05

Bt.28518.1.S1_at			-5.596	1.19E-06
Bt.5154.1.S1_s_at			-5.618	5.33E-06
Bt.29726.1.A1_at			-5.784	1.57E-05
Bt.8796.1.S1_at			-6.355	9.11E-07
Bt.23161.2.A1_at			-7.007	1.33E-07
Bt.26965.1.A1_at			-9.395	1.62E-03
Bt.9122.1.A1_at			-9.663	1.50E-06
Bt.9594.1.S1_at			-9.695	1.39E-09
Bt.19909.1.A1_at			-11.313	7.97E-12
Bt.18855.1.A1_at			-13.418	5.02E-05
