# Supplementary material for: Transcriptome profiling of granulosa cells of bovine ovarian follicles during growth from small to large antral sizes
Source: BMC Genomics. 2014 Jan 14;15:24. doi: 10.1186/1471-2164-15-24 (PMC3898003; doi:10.1186/1471-2164-15-24)
Supplement: Additional file 4: Figure S3 — The complete canonical Axonal Guidance Signalling pathway as presented in IPA showing which genes map from the 3-fold differentially-expressed dataset with a Benjamini-Hochberg FDR multiple correction P < 0.05 between large and small healthy follicles. Genes which are up regulated in large are indicated in red, and those which are down regulated are green, with the degree of fold difference commensurate with the color intensity. [file 1471-2164-15-24-S4.pdf]

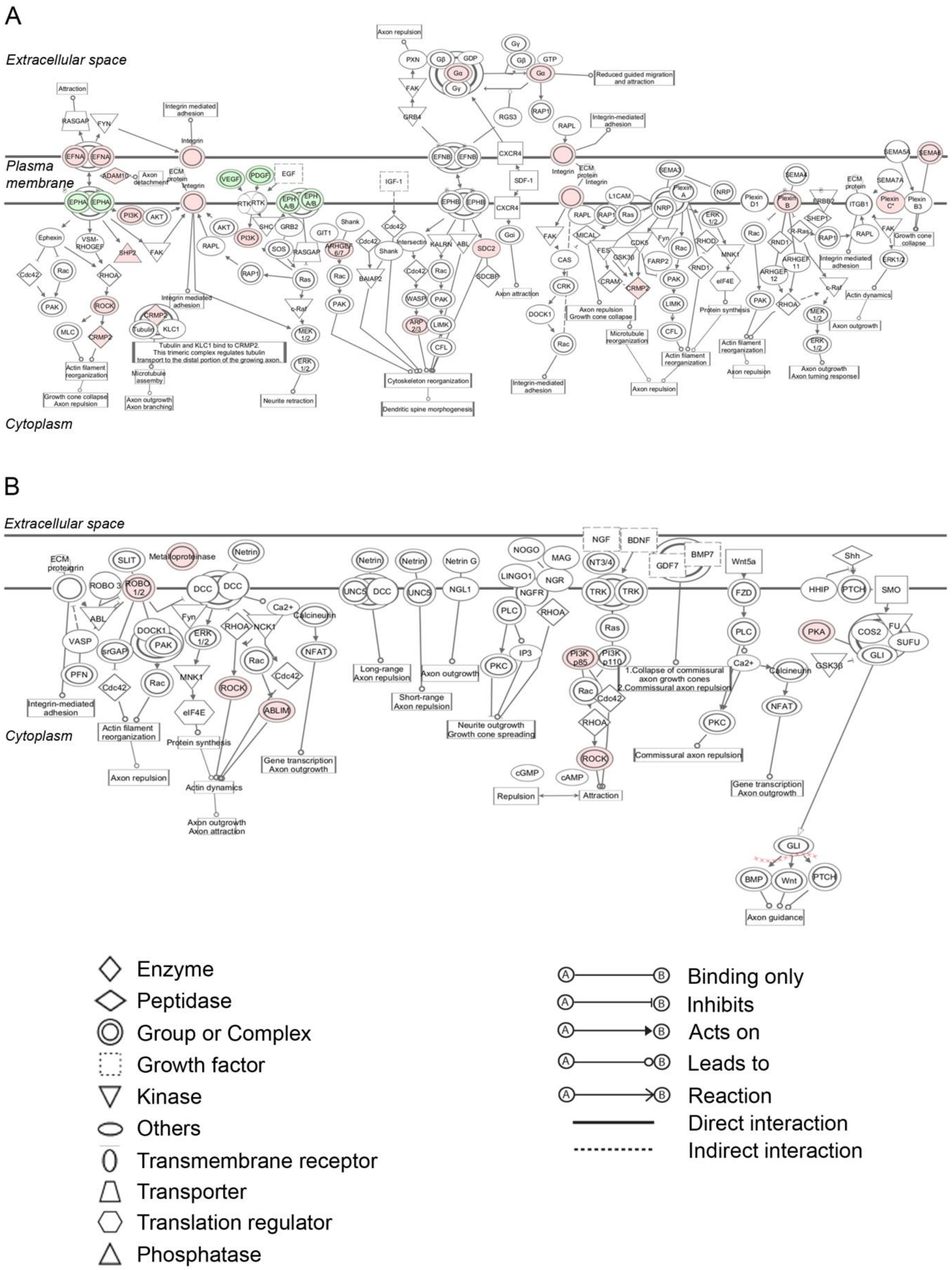
A
Extracellular space
Plasma membrane
Cytoplasm
B
Extracellular space
Cytoplasm
Enzyme
Peptidase
Group or Complex
Growth factor
Kinase
Others
Transmembrane receptor
Transporter
Translation regulator
Phosphatase
Binding only
Inhibits
Acts on
Leads to
Reaction
Direct interaction
Indirect interaction
