# Supplementary material for: Transcriptome profiling of granulosa cells of bovine ovarian follicles during growth from small to large antral sizes
Source: BMC Genomics. 2014 Jan 14;15:24. doi: 10.1186/1471-2164-15-24 (PMC3898003; doi:10.1186/1471-2164-15-24)
Supplement: Additional file 5: Figure S4 — The complete canonical IL-6 signalling pathway as presented in IPA showing which genes map from the 3-fold differentially-expressed dataset with a Benjamini-Hochberg FDR multiple correction P < 0.05 between small and large healthy follicles. Genes which are up-regulated in large are indicated in red, and those which are down-regulated are green, with the degree of fold difference commensurate with the color intensity. [file 1471-2164-15-24-S5.pdf]

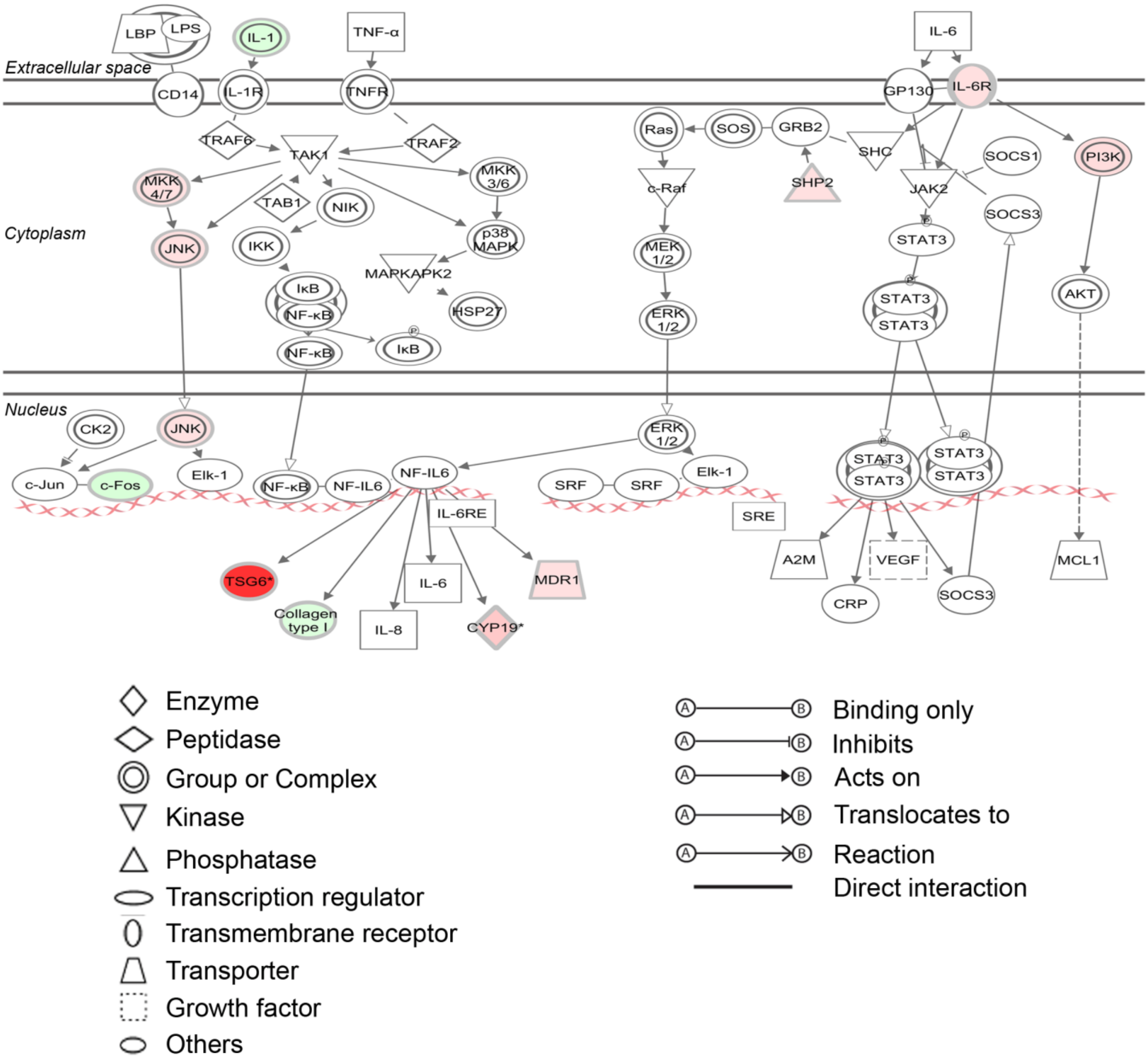
Extracellular space
Cytoplasm
Nucleus
LBP
LPS
IL-1
TNF-α
IL-6
CD14
IL-1R
TNFR
GP130
IL-6R
TRAF6
TAK1
TRAF2
MKK 4/7
TAB1
NIK
MKK 3/6
JNK
IKK
p38 MAPK
MAPKAPK2
IκB
NF-κB
HSP27
Ras
SOS
GRB2
SHC
SHP2
c-Raf
MEK 1/2
ERK 1/2
SOCS1
SOCS3
JAK2
STAT3
PI3K
AKT
CK2
c-Jun
c-Fos
Elk-1
NF-IL6
IL-6RE
SRF
SRE
TSG6*
Collagen type I
IL-8
CYP19*
MDR1
A2M
CRP
VEGF
MCL1
Enzyme
Peptidase
Group or Complex
Kinase
Phosphatase
Transcription regulator
Transmembrane receptor
Transporter
Growth factor
Others
Binding only
Inhibits
Acts on
Translocates to
Reaction
Direct interaction
